# Cellular and Transcriptional Responses of Human Bronchial Epithelial Cells to Delta-9-Tetrahydrocannabinol In Vitro

**DOI:** 10.3390/ijms26115212

**Published:** 2025-05-29

**Authors:** Megan S. Doldron, Sourav Chakraborty, Santosh Anand, Mehwish Faheem, Beh Reh, Xuegeng Wang, Saurav Mallik, Zhenquan Jia, Ramji Kumar Bhandari

**Affiliations:** 1 Department of Biology, University of North Carolina Greensboro, Greensboro, NC 27412, USAx_wang24@uncg.edu (X.W.);; 2 Division of Biological Sciences, University of Missouri, Columbia, MO 65211, USAanand.s@missouri.edu (S.A.);; 3 Harvard Public Health, Department of Environmental Health, Harvard University, Boston, MA 02115, USA; smallik@hsph.harvard.edu

**Keywords:** delta-9-THC, bronchial epithelial cells, transcriptome, in vitro, ferroptosis

## Abstract

Delta-9-tetrahydrocannabinol (Δ-9-THC or THC), the primary psychoactive constituent of cannabis, can lead to adverse health conditions, including mental health issues, brain impairment, and cardiac and respiratory problems. The amount of THC in cannabis has steadily climbed over the past few decades, with today’s cannabis having three times the concentration of THC compared to 25 years ago. Inhalation is a major route of exposure, allowing substances to enter the body via the respiratory tract. THC exposure causes cell death in the airway epithelium; however, the molecular underpinning of THC exposure-induced bronchial epithelial cell death is not clearly understood. To address the mechanisms involved in this process, the present study examined the cell viability, oxidative stress, lipid peroxidation, and transcriptional alterations caused by various concentrations of Δ-9-THC (0, 800, 1000, 1200, and 1500 ng/mL) in a human bronchial epithelial cell line (BEAS-2B) in vitro. Δ-9-THC exposure caused a significant dose-dependent decrease in cell viability after 24 h exposure. Transcriptome analysis showed a distinct dose-dependent response. HIF-1 signaling, ferroptosis, AMPK signaling, and immunogenic pathways were activated by Δ-9-THC-upregulated genes. Glutathione and fatty acid metabolic pathways were significantly altered by Δ-9-THC-dependent downregulated genes. Ingenuity Pathway Analysis (IPA) revealed several top canonical pathways altered by Δ-9-THC exposure, including ferroptosis, NRF-2-mediated oxidative stress response, caveolar-mediated endocytosis (loss of cell adhesion to the substrate), tumor microenvironment, HIF1alpha signaling, and the unfolded protein response pathway. Δ-9-THC-induced cell death was ameliorated by inhibiting the ferroptosis pathway, whereas treatments with ferroptosis agonist exacerbated the cell death process, suggesting that Δ-9-THC-induced bronchial epithelial cell death potentially involves the ferroptosis pathway.

## 1. Introduction

Cannabis, derived from *Cannabis sativa*, is one of the most commonly used illicit drugs in the United States for both medicinal and recreational purposes [1,2]. Although cannabis is one of the oldest multi-usage cultivated crops, its primary use has always been medicinal [3]. This annual herb has gained a lot of attention recently, as many states are legalizing and decriminalizing its use for recreational purposes. Due to the associated health benefits, extensive research is being conducted on the secondary components of cannabis, referred to as cannabinoids [4,5,6]. Cannabis is composed of over 400 different chemical compounds with approximately 66 phytocannabinoids [7]. These phytocannabinoids are naturally occurring oxygen-containing aromatic hydrocarbon compounds found in *Cannabis sativa*.

The common cannabinoids that are active ingredients of cannabis are delta-9-tetrahydrocannabinol (Δ-9-THC) and cannabidiol (CBD). In an epidemiological study, patients who had a cannabis dependence exhibited elevated levels of IL-6 and IL-8, indicating that exposure can induce inflammation in humans [8]. Primary airway epithelial cells exposed to Δ-9-THC showed decreased mitochondrial membrane potential and cell viability, indicating mitochondrial membrane damage [9]. Δ-9-THC, the primary psychoactive constituent, is a partial agonist of the cannabinoid receptors in the endocannabinoid signaling system involved in developing a wide range of biological and behavioral responses [10].

The endocannabinoid system (ECS) is a complex and versatile signaling network that plays a crucial role in maintaining homeostasis within the human body. The ECS is involved in a wide array of physiological processes, including pain modulation, appetite regulation, immune response, and neuroprotection, highlighting its significance in both health and disease [11,12,13]. Endogenous cannabinoid receptors are part of a larger unit called the endocannabinoid system, which is composed of cell-surface G-protein-coupled cannabinoid CB1 and CB2 receptors that are known to regulate cellular processes such as cell survival, cell death, and cell proliferation [14]. CB1 receptors are located throughout the body and can be found in the brain, liver, kidneys, ovaries, and lungs [15]. The lungs are a major route of exposure, as inhalation allows substances to enter the body and interact with the epithelial components of the respiratory tract. Previous studies have shown an increase in the prevalence of symptoms indicative of a wide variety of respiratory diseases and conditions, such as cough, shortness of breath, acute bronchodilation, wheezing, and airway obstruction due to cannabis exposure [16,17,18]. Some of these respiratory diseases were associated with an elevated level of oxidative stress [19]. Δ-9-THC increases hydrogen peroxide production, which can also lead to oxidative stress levels [19]. Oxidative stress occurs due to the overproduction of reactive oxygen species (ROS). The accumulation of ROS can initiate cell death involving the ferroptosis pathway, which is strongly correlated with the progression of many lung diseases [20,21]. Ferroptosis has been found to be a pathway involved in various forms of cell death, distinct from apoptosis and necrosis, and is increasingly recognized for its role in cancer and neurodegenerative diseases [22]. In BEAS-2B cells, cigarette smoke exposure, which induces oxidative stress, resulted in decreased glutathione levels and increased ferroptosis markers [23], and the increased ROS levels led to lipid peroxidation and the depletion of glutathione, thereby promoting ferroptosis [24]. In bone marrow-derived macrophages, Δ-9-THC increased cellular iron levels and ROS, indicating a possible modulation of ferroptosis sensitivity [25]. It is not clearly understood if Δ-9-THC can induce cell death via ferroptosis in the bronchial epithelium.

According to the Substance Abuse and Mental Health Services Administration (SAMHSA, https://www.samhsa.gov, accessed on 25 August 2024) of the U.S. Department of Health and Human Services, the amount of Δ-9-THC in cannabis has steadily increased over the past few decades, with today’s cannabis having three times the concentration of Δ-9-THC compared to that seen 25 years ago [26]. Given the higher concentration of Δ-9-THC and increased use of cannabis for medicinal and recreational purposes, it is imperative to understand the molecular underpinnings of Δ-9-THC exposure in the lung epithelium, as lung cells receive significant amounts of Δ-9-THC during cannabis smoking before it reaches other parts of the body via the circulation. How cells respond to various concentrations of Δ-9-THC and what mechanisms are involved in mediating its effects in the lungs are not clearly understood. The present study aims to identify transcriptional alterations induced by THC in normal human bronchial cells. We assessed dose responses of Δ-9-THC exposure using human bronchial epithelial cells (BEAS-2B) in vitro and examined transcriptional responses of bronchial epithelial cells by RNA sequencing followed by Gene Ontology enrichment (GO) and pathway analyses. The pathways identified by this comprehensive analysis provide insights for further understanding the mechanism of Δ-9-THC-induced toxicity in human lungs and reveal avenues for further research to design a mitigation strategy.

## 2. Results

### 2.1. Δ-9-THC Dose-Dependent Viability of BEAS-2B Cells

**Figure 1:**
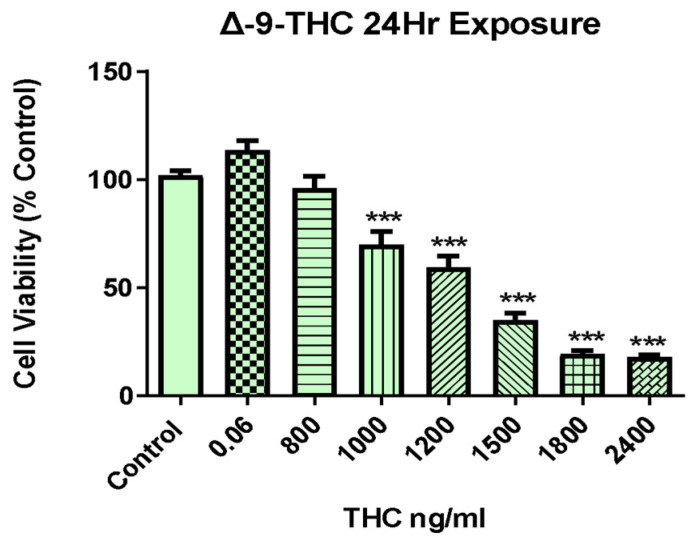
Effect of THC on BEAS-2B growth parameters. Cell viability was assessed after 24 h of Δ-9-THC (0.06, 800, 1000, 1200, 1500, 1800, 2400 ng/mL) exposure followed by MTT analysis for three replicates. Data represent mean ± SEM. Statistical significance *** *p* < 0.001.

In order to determine cell viability, a 3-(4,5-dimethylthiazol-2-Yl)-2,5-diphenyltetrazolium bromide (MTT) cell proliferation assay was conducted on BEAS-2B cells upon Δ-9-THC exposure. As illustrated in Figure 1, BEAS-2B cells treated with varying concentrations of Δ-9-THC (0.06, 800, 1000, 1200, 1500, 1800, and 2400 ng/mL) for 24 h showed decreased cell viability compared with that in the control group in a dose-dependent manner. The medium control, vehicle control, and 800, 1000, 1200, and 1500 ng/mL Δ-9-THC groups were selected for RNA sequencing to find novel and conserved molecular pathways related to cell death.

### 2.2. Δ-9-THC Dose-Mediated Global Alterations in Gene Expression Profile, Biomarkers, Transcription Factors, and Canonical Pathways

**Figure 2:**
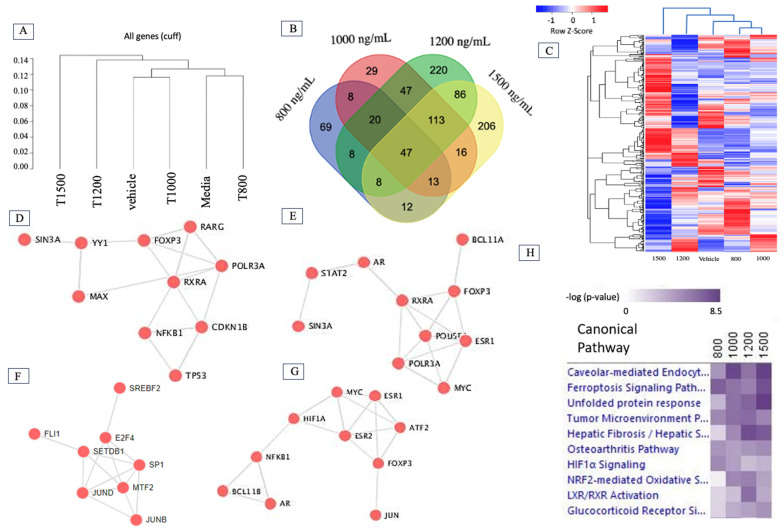
Global transcriptional alteration in BEAS-2B cells by Δ-9-THC. (**A**) Hierarchical relationship of differential gene expression at varying dosage concentrations of Δ-9-THC (medium control; vehicle control; 800, 1000, 1200, and 1500 ng/mL). (**B**) Venn diagram showing highly significant DEGs in all treatment groups and their overlap among groups, suggesting a dose-dependent response in gene expression profile. (**C**) A heatmap showing all DEGs in BEAS-2B cells in response to exposure to various concentrations of THC for 24 h. List of transcription factors associated with Δ-9-THC dosing concentrations of (**D**) 800 ng/mL, (**E**) 1000 ng/mL, (**F**) 1200 ng/mL, and (**G**) 1500 ng/mL. (**H**) Ingenuity Pathway Analysis revealed canonical pathways activated by Δ-9-THC in BEAS-2B cells, suggesting an association with the ferroptosis signaling pathway across the dosing concentrations of Δ-9-THC.

Ingenuity Pathway Analysis ^®^ (IPA ^®^ v. 9.0) and R scripts were used to determine the pathways related to differentially expressed genes (DEGs) in the groups treated with different concentrations of Δ-9-THC. To elucidate the closeness of DEGs mediated by Δ-9-THC, a dendrogram was plotted. The results showed that DEGs found in groups treated with 800 ng/mL and medium were clustered together, whereas those in the 1200 ng/mL and 1500 ng/mL treatment groups were far from the other experimental groups (Figure 2A). The most significant number of overlapping DEGs was found in the groups treated with 1000 ng/mL, 1200 ng/mL, and 1500 ng/mL Δ-9-THC (Figure 2B). The global alterations in gene expression profiles associated with the Δ-9-THC treatment groups are presented in Figure 2C and Supplementary Figure S1. Not only the DEGs but also their expression patterns were similar between the vehicle control and 800 ng/mL Δ-9-THC groups, suggesting that low-concentration Δ-9-THC did not cause any changes in BEAS-2B cells. Transcriptomic biomarkers were identified from RNAseq datasets using −2 < Log2FC > 3 and FDR 0.05. MMP1 and IL1RL1 were significantly upregulated in the 800 ng/mL and 1000 ng/mL Δ-9-THC groups compared to the vehicle controls (Supplementary Table S1). However, HSPA1A, HSPA1B, HSPA6B, and HMOX were identified as biomarkers in the 1200 and 1500 ng/mL Δ-9-THC-treated groups (Supplementary Table S1). To determine the specific transcription factors associated with aberrant gene expression, transcription factor networks were analyzed. Several genes, mainly RARG, FOXP3, RXR1A, TPS3, and NFKB1 were found to be markers in the 800 ng/mL group (Figure 2D), whereas RXRA, POLR3A, AR, MYC, ESR1, STAT2, and FOXP3 were associated with the 1000 ng/mL Δ-9-THC group (Figure 2E). In addition, SREBF2 (SREBP), SETDB1, SP1, JUND, MTF2, E2F4, and FLI2 were the markers in the 1200 ng/mL group, while ESR1, ESR2, NFKB1, AR, JUN, MYC, HIF1A, FOXP3 (Figure 2F), and ATF2 were found to be associated with the 1500 ng/mL Δ-9-THC group (Figure 2G). Ferroptosis signaling, caveolar-mediated endocytosis, and tumor microenvironment pathways were activated across all Δ-9-THC treatment groups. The NRF2-mediated oxidative pathway and LXR/RXR activation pathway were highlighted in the 1000 ng/mL, 1200 ng/mL, and 1500 ng/mL Δ-9-THC groups (Figure 2H).

### 2.3. Δ-9-THC-Mediated Enrichment of Long Non-Coding RNA (lncRNA) in BEAS-2B Cells Associated with Activation of Lung Cancer Pathways

**Figure 3:**
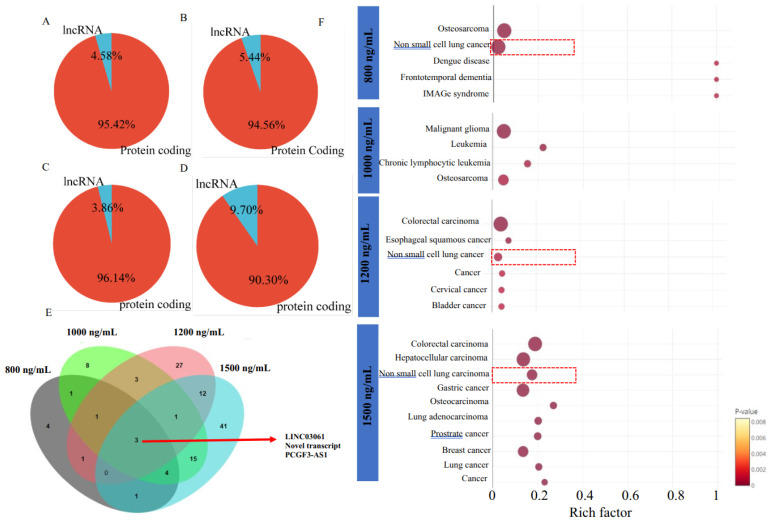
Association of long non-coding RNAs linked to Δ-9-THC dose responses. Percentage of lncRNAs and protein-coding genes found in the groups treated with (**A**) 800 ng/mL, (**B**) 1000 ng/mL, (**C**) 1200 ng/mL, and (**D**) 1500 ng/mL Δ-9-THC. (**E**) Venn diagram showing highly significant long non-coding RNAs (lncRNAs) in all treatment groups and their overlap. (**F**) Enriched pathways associated with lncRNAs in each treatment group. Exposure to Δ-9-THC at concentrations of 800, 1200, and 1500 ng/mL was found to be associated with the activation of the non-small cell lung cancer (NSCLC)-linked signaling pathway, potentially mediated through the dysregulation lncRNAs. Red circles represent the gene ratios associated with various diseases, with the area of each circle proportional to the corresponding gene ratio. Red dashed boxes highlight key diseases significantly enriched with lncRNAs.

Global gene expression analysis identified several long non-coding RNAs (lncRNAs) activated by Δ-9-THC exposure. The percentage of lncRNA relative to protein-coding genes was 4.58%, 5.44%, 3.86%, and 9.70% in the 800 ng/mL, 1000 ng/mL, 1200 ng/mL, and 1500 ng/mL Δ-9-THC groups, respectively (Figure 3A–D). The Venn diagram displays the distribution of lncRNAs across different treatment groups (Figure 3E). Three common lncRNAs were identified across all dosing concentrations of Δ-9-THC. The lncRNA pathway enrichment analysis identified non-small cell lung cancer-related species in the 800 ng/mL, 1200 ng/mL, and 1500 ng/mL Δ-9-THC groups (Figure 3F). The lncRNA identified in the 1000 ng/mL group was associated with malignant glioma development.

### 2.4. HIF-1 Signaling, Ferroptosis, AMPK Signaling, and Immunogenic Pathways Were Enriched in Genes Dose-Dependently Upregulated by Δ-9-THC in BEAS-2B Cells

**Figure 4:**
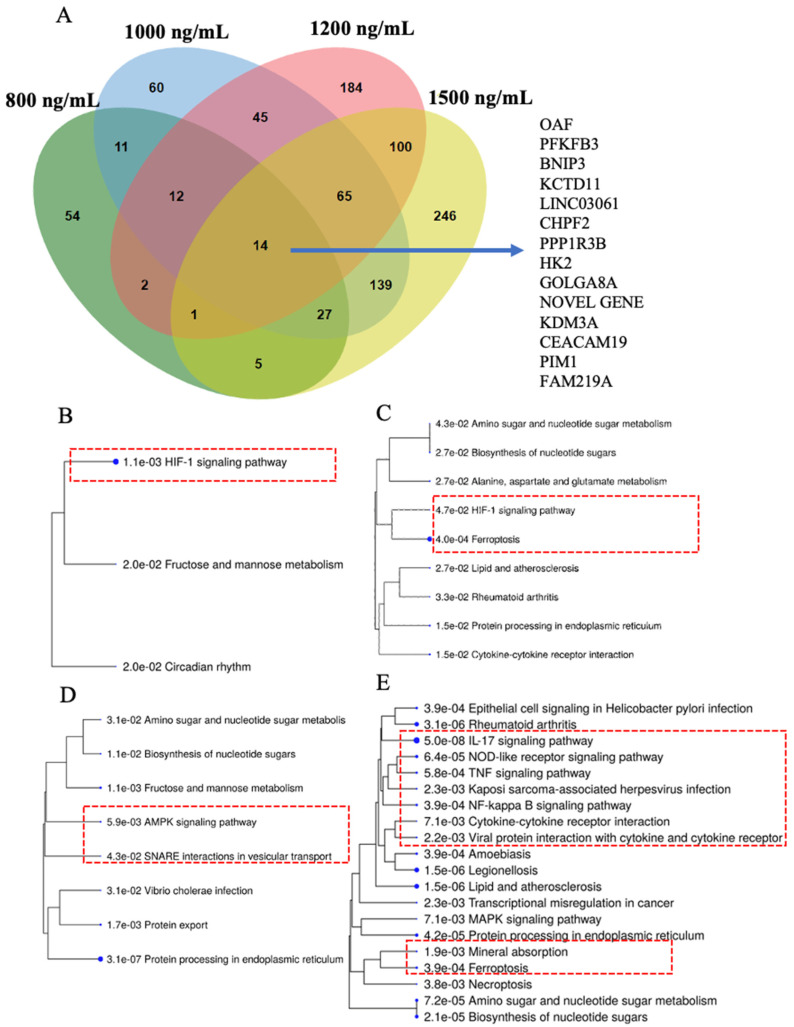

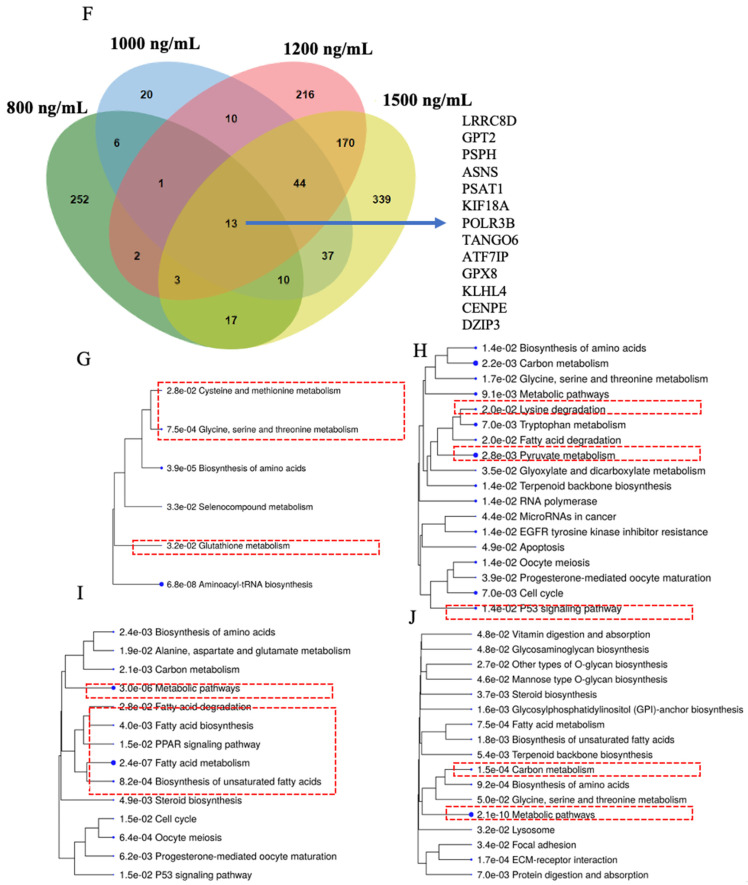
Δ-9-THC-dose-dependently upregulated genes and their association with pathways. (**A**) Venn diagram showing the overlap of upregulated DEGs associated with Δ-9-THC and fourteen genes common to all dosing concentrations of Δ-9-THC. Upregulated DEG-driven pathways in the (**B**) 800 ng/mL, (**C**) 1000 ng/mL, (**D**) 1200 ng/mL, and (**E**) 1500 ng/mL Δ-9-THC treatment groups. Δ-9-THC-dose-dependently downregulated genes and their association with pathways. (**F**) Venn diagram showing the overlap of downregulated DEGs associated with Δ-9-THC exposure in vitro and thirteen genes common to all dosing concentrations of Δ-9-THC. Downregulated DEG-driven pathways in the (**G**) 800 ng/mL, (**H**) 1000 ng/mL, (**I**) 1200 ng/mL, and (**J**) 1500 ng/mL Δ-9-THC treatment groups. Red dashed boxes indicate significant biological pathways linked to Δ-9-THC treatment.

To find common and distinctively expressed upregulated genes in BEAS-2B cells across all Δ-9-THC treatment groups, all upregulated genes were plotted in a Venn diagram. A total of fourteen genes were found to be commonly upregulated at all experimental dosing concentrations of Δ-9-THC (Figure 4A), and those genes were associated with the activation of HIF1 signaling, multiple glucose metabolism, and metabolic pathways (Supplementary Figure S2A). Further, 100 shared genes were significantly upregulated in the 1200 ng/mL and 1500 ng/mL Δ-9-THC-treated groups (Figure 4A). Genes upregulated in the 800 ng/mL and 1000 ng/mL Δ-9-THC groups compared to the vehicle control were associated with activating the HIF-1 pathway (Figure 4B), while the ferroptosis pathway was enriched in upregulated genes in the 1000 ng/mL and 1500 ng/mL Δ-9-THC groups (Figure 4C,E). AMPK signaling was found to be triggered in the 1200 ng/mL Δ-9-THC group (Figure 4D). Furthermore, in the 1500 ng/mL group, most immunogenic pathways, such as NF-kappa B signaling, TNF signaling, NOD-like receptor signaling, cytokine–cytokine receptor interaction, and IL-17 signaling, were activated by upregulated genes (Figure 4E). The results indicated that upregulated genes in BEAS-2B cells were linked to disease-specific molecular pathways, including stress, immunogenic, and carcinogenic pathways.

### 2.5. Cysteine–Methionine Metabolism, Glutathione Metabolism, Amino Acid Metabolism, and Fatty Acid Metabolism Were Significantly Enriched in THC-Mediated Downregulated Genes

Among all downregulated genes, 13 genes were commonly downregulated across all Δ-9-THC treatment groups compared to their expression in the vehicle control (Figure 4F). Carbon metabolism; alanine, aspartate, and glutamate metabolism; glycine, serine, and threonine metabolism; and arginine biosynthesis were enriched by shared downregulated genes (Supplementary Figure S2B) across all treatment groups. Downregulated genes found in the 800 ng/mL treatment group were associated with cysteine and methionine metabolism and glutathione metabolism (Figure 4G). In the 1000 ng/mL treatment group, p53 signaling, pyruvate metabolism, and lysine degradation pathways were enriched by downregulated genes (Figure 4H). Furthermore, fat metabolism linked to fatty acid degradation, fatty acid biosynthesis, and PPAR signaling pathways were significantly dysregulated by downregulated genes in the 1200 ng/mL treatment group (Figure 4I). In the 1500 ng/mL treatment group, focal adhesion, metabolic pathways, lysosome, and O-glycan biosynthesis were significantly altered by downregulated genes (Figure 4J).

### 2.6. Functional Network Analysis and Enrichment of Mammalian Phenotypes Associated with DEGs

**Figure 5:**
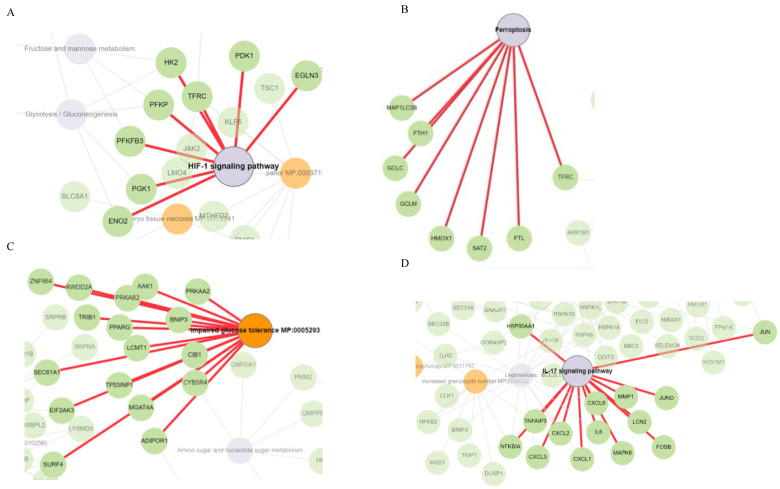
Δ-9-THC-dose-dependent pathways and associated genes found in disease-specific pathways. Genes associated with the (**A**) HIF-1 signaling pathway triggered in the 800 ng/mL group, (**B**) ferroptosis pathway induced by 1200 ng/mL Δ-9-THC, (**C**) impaired glucose tolerance in the 1500 ng/mL group, (**D**) and IL17 signaling pathways in the 1500 ng/mL treatment group.

To investigate the molecular effects of DEGs on human lung epithelial cells due to Δ-9-THC, Gene Ontology (GO) analysis was performed to find molecular function (MF), biological process (BP), and cellular component (CC) pathways. The biological pathways driven by DEGs in BEAS-2B cells, such as response to hypoxia (*p* value 2.408 × 10^−4^) and monosaccharide metabolic process (*p* value 1.004 × 10^−3^), were significantly altered in the 800 ng/mL groups (Supplementary Figure S3A). In the 1000 ng/mL treatment group, protein binding (MF, *p* value 1.98 × 10^−11^), programmed cell death (BP, *p* value 1.455 × 10^−6^), autophagy, organelle disassembly (BP, *p* value 9.64 × 10^−3^), ubiquitin-dependent ERAD pathway (BP, *p* value 3.45 × 10^−3^), and endoplasmic reticulum chaperon complex (CC, *p* value 2.96 × 10^−3^) were significantly altered (Supplementary Figure S3B). 2-oxoglutarate-dependent dioxygenase activity (MF, *p* value 3.9 × 10^−10^), cellular response to stress (BP, *p* value 3.22 × 10^−3^), and autophagy (BP, *p* value 3.71 × 10^−2^) were found to be altered by 1200 ng/mL Δ-9-THC (Supplementary Figure S3C). In the group exposed to the highest concentration of Δ-9-THC (1500 ng/mL), protein folding chaperone (MF, *p* value 1.533 × 10^−5^), response to unfolded protein (BP, *p* value 9.056 × 10^−18^), cellular stress response (BP, *p* value 4.94 × 10^−13^), and inflammatory response (BP, *p* value 1.33 × 10^−3^) were significantly enriched by DEGs. GO results indicated that Δ-9-THC-dose-dependent disease-specific pathways were triggered. In the cells exposed to the lowest concentration of Δ-9-THC (800 ng/mL), genes such as ENO2, PGK1, and HK2 were significantly upregulated and associated with HIF-1 signaling (Figure 5A), glycogen biosynthesis, and abnormal muscle fiber morphology as mammalian phenotypes (Supplementary Figures S4 and S5). MAP1LC3B, FTH1, GCLC, GCLM, HMOX, SAT2, and FTL were associated with the activation of the ferroptosis pathway due to 1000 ng/mL of Δ-9-THC exposure (Figure 5B). Furthermore, a significant number of genes such as TPP1, GFPT1, HSPA5, SERP1, and HYOU1 were associated with the IRE1-mediated unfolded protein response, endoplasmic stress response, and impaired glucose tolerance pathways; decreased circulating insulin levels were associated with 1000 ng/mL Δ-9-THC exposure. (Supplementary Figures S6 and S7). Impaired glucose tolerance (Figure 5C), endoplasmic reticulum to Golgi vesicle-mediated transport, SNARE interaction in vesicular transport, and impaired glucose tolerance were found to be enriched by a significant number of genes triggered by 1200 ng/mL Δ-9-THC (Supplementary Figure S8). Abnormal circulating protein levels and decreased cellular hemoglobin content were linked with 1200 ng/mL Δ-9-THC treatment (Supplementary Figure S9) in BEAS-2B cells. The highest Δ-9-THC (1500 ng/mL) concentration was found to activate a significant number of immunogenic genes such as CXCL 2 and 3, IL6, MMP1, and MAPK6 with the activation of the IL17 signaling pathway (Figure 5D), as well as common DEGs enriched in the oxidative stress pathway and response to endoplasmic reticulum stress (Supplementary Figure S10).

### 2.7. Aberrant Expression of Ferroptosis, Autophagy, and ER Response Genes

**Figure 6:**
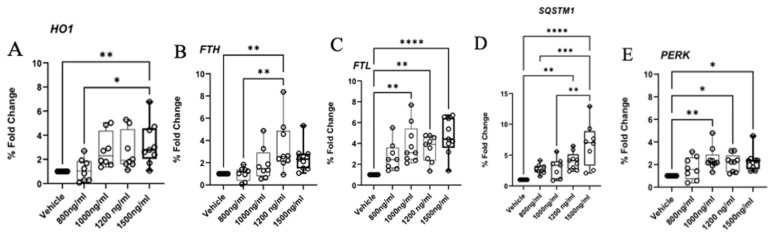
Validation of the RNAseq results by RT-qPCR. Relative expression levels of the DEGs between the bronchial cell groups exposed to different concentrations of Δ-9-THC. (**A**) HO1, (**B**) FTH, (**C**) FTL, (**D**) SQSTM1, (**E**) PERK genes showed dose specific Δ-9-THC response. Asterisks indicate statistical significance (* *p* < 0.05, ** *p* < 0.01, *** *p* < 0.001, **** *p* < 0.0001).

To validate the RNAseq gene expression data, we selected several upregulated targets and examined their transcript levels by RT-qPCR. A significant upregulation of the gene encoding essential pro-ferroptosis enzyme HO1 (Figure 6A) and the gene encoding iron storage proteins FTH and FTL (Figure 6B,C) were observed. Furthermore, a significant upregulation of SQSTM1 (Figure 6D) and PERK (Figure 6E) in response to autophagy and ER stress, respectively, was also observed. Gene expression patterns for HO1, FTH, FTL, and SQSTM in the 1200 ng/mL Δ-9-THC group after treatment with Fer-1, ERA, and a combination of ERA and Fer-1 was examined, and the relative fold change was insignificant.

### 2.8. Cell Viability and Ferroptosis

**Figure 7:**
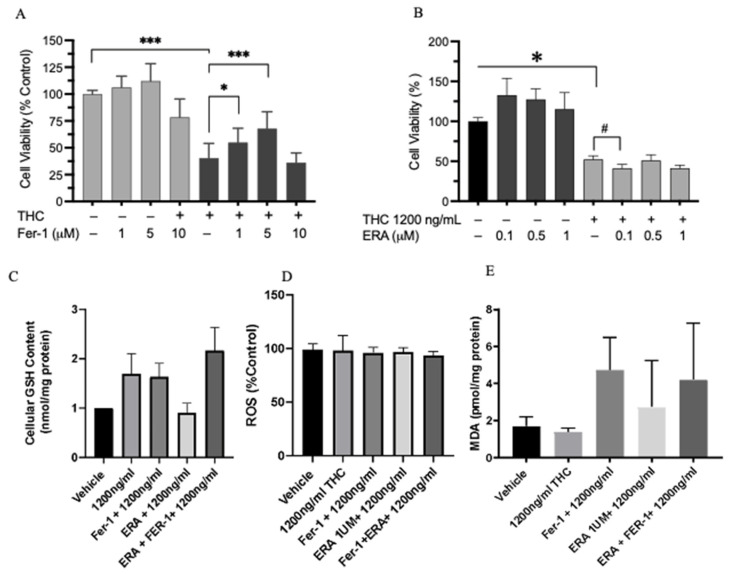
Confirmation of the involvement of ferroptosis in Δ-9-THC-induced cell death in vitro. Effects of Δ-9-THC (1200 ng/mL) together with the ferroptosis antagonist Fer-1 (**A**) or the agonist ERA (**B**). Data represent ± SEM. Statistical significance *** *p* < 0.001, * *p* < 0.05, # *p* < 0.06 (n = 3). Effects of THC with or without a ferroptosis agonist or antagonist on GSH content (**C**), ROS level (**D**), and lipid peroxidation (**E**). Data represent ± SEM. Statistical significance *** *p* < 0.001 (n = 3 independent assays).

To characterize the role of ferroptosis in Δ-9-THC-treated cells, the cells were treated with 1200 ng/mL Δ-9-THC with and without the ferroptosis inhibitor Fer-1 or agonist ERA for 24 h. Δ-9-THC treatment at this concentration significantly decreased cell viability. Treatment with Fer-1 at 5 μM increased cell viability in the Δ-9-THC-exposed cells but not the control (Figure 7A). In contrast, the ferroptosis agonist ERA caused a further reduction in cell viability in Δ-9-THC-exposed cells but only at 0.1 μM (Figure 7B). At the higher concentrations (0.5 and 1 μM) there was no significant effect. Overall, these results suggest that the ferroptosis pathway is at least partially involved in the response of BEAS-2B cells to Δ-9-THC exposure. Our data showed that BEAS-2B cells exposed to Δ-9-THC + Fer-1, ERA + Δ-9-THC, and Δ-9-THC + Fer-1 + ERA did not exhibit a significant change in cellular GSH levels, as seen in Figure 6C. However, there was a slight decrease in the viability of cells cotreated with ERA + Δ-9-THC (Figure 7C). To determine if there was a significant increase in reactive oxygen species due to Δ-9-THC-induced cell death, cells were treated with Δ-9-THC or a ferroptosis inhibitor and ferroptosis agonist. As shown in Figure 7D, ROS levels were not altered in any of the conditions compared to the vehicle control. The levels of MDA, an indicator of lipid peroxidation, were measured following a 24 h cotreatment of Δ-9-THC + ERA and a 1 h pretreatment of Fer-1 followed by a 24 h exposure to Δ-9-THC. Δ-9-THC exposure did not significantly increase MDA levels (Figure 7E). There was no significant difference in MDA levels in cells exposed to THC together with the inhibitor Fer-1 and the agonist ERA.

## 3. Discussion

The present study investigated the impact of 24 h Δ-9-THC exposure on the transcriptional profiles of bronchial epithelial cells by RNA sequencing. Bronchial epithelial cells exposed to Δ-9-THC concentrations less than the human relative concentration exhibited cell death. Key observations in this study included decreased dose-dependent cell viability, activation of the ferroptosis pathway, and the enrichment of long non-coding RNAs that are implicated in lung cancer. In human lung cancer cells, Δ-9-THC caused a dose-dependent decrease in mitochondrial membrane potential [27]. Decreased mitochondrial membrane potential enhances the increased production of ROS and apoptotic cell death [28,29]. Several key canonical pathways affected by THC exposure included ferroptosis, the tumor microenvironment, and the unfolded protein response. In addition, the significant enrichment of long non-coding RNAs (lncRNAs) at different Δ-9-THC concentrations highlights a potential link between THC exposure and lung carcinogenesis. The presence of lncRNAs associated with non-small cell lung cancer (NSCLC) at multiple THC doses and with malignant glioma at 1000 ng/mL suggests that Δ-9-THC may influence cancer-associated pathways through epigenetic mechanisms.

Hierarchical clustering and dendrogram analysis of DEGs in groups treated with different doses of THC revealed that DEGs in the 800 ng/mL Δ-9-THC treatment group were similar to those in the vehicle and medium controls, whereas DEGs from the 1200 ng/mL and 1500 ng/mL groups clustered together, indicating a distinct transcriptional response at higher concentrations. The Venn diagram further supported this observation, showing a significant overlap of DEGs in the 1200 ng/mL and 1500 ng/mL Δ-9-THC groups, suggesting a shared molecular response to high-dose exposure. At lower concentrations (800 ng/mL and 1000 ng/mL), significant upregulation of MMP1 and IL1RL1 was observed, indicating early inflammatory responses. Our findings demonstrate that Δ-9-THC induces significant molecular alterations in human lung epithelial cells (BEAS-2B) in a dose-dependent manner. At lower concentrations (800 ng/mL), genes such as ENO2, PGK1, and HK2 were significantly upregulated, indicating the activation of the HIF-1 signaling pathway. This suggests that Δ-9-THC exposure at this level may lead to metabolic reprogramming and cellular adaptations to hypoxic conditions. The overexpression of HIF1-alpha has been associated with lung cancer and is correlated with cigarette smoke [30]. Specifically, genes linked to the HIf1-alpha signaling pathway are shown to have a significant impact on lung carcinogenesis and genomic instability [31]. Moreover, the activation and overexpression of the HIf1-alpha signaling pathway have also been linked to COPD and decreased lung function [32]. Exposure to 1200 ng/mL Δ-9-THC further reinforced the role of oxidative stress and metabolic disruption, with alterations observed in 2-oxoglutarate-dependent dioxygenase activity and cellular response to stress. The enrichment of endoplasmic reticulum (ER) to Golgi vesicle-mediated transport and SNARE interactions in vesicular transport suggests that Δ-9-THC may impair intracellular trafficking, potentially affecting protein secretion and cellular communication. At the highest concentration (1500 ng/mL), we observed significant immune activation, with the upregulation of CXCL2, CXCL3, IL6, MMP1, and MAPK6, which are associated with the IL-17 signaling pathway. This suggests that Δ-9-THC at high doses may induce a pro-inflammatory state, potentially contributing to lung inflammation and immune dysregulation. Additionally, genes enriched in oxidative stress pathways and response to ER stress indicate that cellular stress responses are significantly triggered at this concentration, which could lead to chronic inflammation and tissue damage.

Notably, the ferroptosis signaling pathway, the unfolded protein response, and caveolar-mediated endocytosis were commonly triggered across all treatment groups. In addition, NRF2-mediated oxidative stress pathways and LXR/RXR activation were observed at Δ-9-THC doses of 1000 ng/mL and 1200 ng/mL, implicating an imbalance in cellular homeostasis and lipid metabolism. When a cell undergoes the unfolded protein response, an increase in the expression of PERK can be expected, as it is one of the primary signal transducers of the UPR pathway. This was displayed in our study, which showed an increase in PERK expression as a result of bronchial epithelial cell exposure to Δ-9-THC. The ferroptosis and unfolded protein response pathways are connected, as half of the lipids in cells are present in the endoplasmic reticulum, suggesting the ER’s role in the initiation of ferroptosis [33,34]. PERK is a known mediator of ferroptosis, as it interacts with NRF2, causing phosphorylation and increasing levels of HMOX1, thus activating ferroptosis [35]. The unfolded protein response is also known to be activated and upregulated in the tumor microenvironment, which is a pathway that was upregulated in the present study [35,36]. The tumor microenvironment is the environment that surrounds the tumor and consists of surrounding blood. The LXR/RXR activation pathway also has a negative impact on lung function [37]. The LXR/RXR activation pathway was slightly enriched in cells exposed to various concentrations of Δ-9-THC. This pathway regulates cholesterol homeostasis and lipid and carbohydrate metabolism [38,39]. When cells die, there is typically an increase in lipid production in which the LXR/RXR pathway breaks down and removes the lipid surplus [38,40]. The LXR/RXR activation pathway also can induce airway inflammation and airway hyperresponsiveness, as an agonist against the LXR/RXR activation pathway can attenuate airway inflammation, asthma, and pulmonary emphysema in vivo in murine models [41].

Ferroptosis is a cell death process that is iron dependent and characterized by lipid peroxidation. This process is mediated by phospholipid peroxidation in association with free-iron-mediated Fenton reactions [20,42]. A correlation between Δ-9-THC exposure and cell death has been demonstrated; however, iron-dependent cell death has not been demonstrated yet. In a study with BEAS-2B cells, ferroptosis was found to be one of the major pathways affected by exposure to cigarette smoke condensate [43]. Another study that exposed airway cells to cigarette smoke showed the upregulation of HMOX1, as seen in our study [44]. HMOX1, a widely used biomarker for oxidative stress, plays a critical role in ferroptosis, as it processes heme and releases Fe^2+^ [44]. This was highlighted in a study that used EF24 on osteosarcoma cells and determined cell death, in which ferroptosis was mediated by HMOX1 [45]. When HMOX1 was upregulated, it resulted in ferroptosis in the osteosarcoma cells and had the exact opposite effect when HMOX1 was knocked down, thus showing its involvement in ferroptosis. This corresponds with the upregulation of HMOX1 in the present study, suggesting that ferroptosis occurred as a result of Δ-9-THC exposure. The deregulation of ferroptosis has been linked to chronic obstructive pulmonary disease (COPD) in airway cells exposed to cigarette smoke extract [23,46]. Despite ferroptosis, a number of other interesting findings in our study aligned with COPD pathogenesis, including the aberrant regulation of glycogen biosynthesis, glycolysis, gluconeogenesis, impaired glucose tolerance, and abnormal muscle fiber morphology. Although glycogen is synthesized by the liver and skeletal muscle, chronic COPD patients experience muscle weakness and inadequate cellular energy supply linked with disturbances in glucose metabolism [47]. Taken together, our results suggest that Δ-9-THC exposure can cause ferroptosis and abnormal glucose metabolism, which may play a role in COPD pathogenesis.

Another key component of ferroptosis is ferritin, which is a major intracellular iron storage protein that is involved in intracellular iron homeostasis. Ferritin is a heteropolymer that includes FTL1 (ferritin light polypeptide 1) and FTH1 (ferritin heavy polypeptide 1), which protect cells from iron-mediated damage [48]. In the present study, we saw an increase in both *FTH1* and *FTL1* expression because of exposure to Δ-9-THC. However, a study by Hou et al. showed a decrease in FTH levels in DHA-exposed leukemia cells as a result of the degradation of ferritin by autophagy, leading to an increase and accumulation of free iron [49]. In contrast, as in our study, FTL expression was increased in RAW264.7 cells that were exposed to LPS [50]. In addition to being an iron storage molecule, FTL is also known to be an antioxidant. The significant increase in FTL1 gene expression observed in the present study may be the result of oxidative stress, specifically lipid peroxidation that may occur because of Δ-9-THC exposure. It is reasonable to assume that if the level of lipid peroxidation increases, the FTL and FTH protein levels may increase, as shown in our study, in a protective manner. Similar to our study, HeLa cells that were exposed to DOX exhibited an increase in ferritin mitochondrial expression to reduce the cytotoxicity that occurred as a result [51].

It was previously reported that BEAS-2B airway cells, when exposed to Δ-9-THC, resulted in cytotoxicity, oxidative stress, and cell death [52,53]. To further evaluate the mechanism behind cell death and the involvement of ferroptosis in Δ-9-THC-induced cytotoxicity, BEAS-2B cells were treated with Fer-1 (5 µM) to ameliorate Δ-9-THC-induced effects. When airway cells were pretreated with Fer-1 for 1 h and then immediately treated with Δ-9-THC for 24 h, there was a significant increase in cell viability compared to that observed after treatment with Δ-9-THC alone. These observations supported previous studies suggesting that Fer-1 prevents oxidative lipid damage and ferroptosis in auditory hair, neuronal, and airway cells [54,55,56]. Ferroptosis is a form of non-apoptotic cell death primed by lipid peroxidation. Fer-1 is a potent ferroptosis inhibitor that prevents lipid peroxidation. Recent studies have shown that Fer-1 has ROS scavenging capabilities, particularly for scavenging alkoxyl radicals [57]. An increase in MDA levels is an indication of lipid peroxidation, which is the major process occurring during ferroptosis. The observation of no significant increase in MDA levels following Δ-9-THC treatment makes the conclusion difficult. Biological processes are multifactorial. The observation of Fer-1 (a ferroptosis inhibitor) significantly rescuing Δ-9-THC -induced cell death suggests that Fer-1-induced blockage of cell death may be mediated via action on other pathways involved in ferroptosis, as the present RNAseq data suggest that Δ-9-THC exposure activated the ferroptosis pathway along with several other pathways, such as endocytosis, unfolded protein response, HIFα signaling, etc. Erastin is an inducer of ferroptosis, as it inhibits cystine import, thus depleting GSH and decreasing GPX4 expression. GPX4 is a key regulator of ferroptosis, as it mediates lipid peroxidation. In this study, when airway cells were cotreated with Δ-9-THC and ERA for 24 h, there was a noticeable decrease in cell viability when exposed to Δ-9-THC and ERA, but this was not observed for ERA alone. Ferroptosis may require its pathway to be activated by a primary stressor, such as Δ-9-THC, since Fer-1 (a ferroptosis inhibitor) significantly rescued Δ-9-THC-induced cell death and low-concentration ERA (ferroptosis agonist) exacerbated the effect. Similar to our results, human pancreatic, neuroblastoma, and airway cells, when exposed to ERA exhibited an increase in cell death and ferroptosis [58,59]. The visible decrease in the viability of BEAS-2B cells cotreated with ERA (0.1 µM) and Δ-9-THC and the significant increase in viability when pretreated with Fer-1 indicate the potential role of ferroptosis in Δ-9-THC-induced cytotoxicity, although many other pathways may be involved in this process, as indicated by the current RNAseq data.

## 4. Materials and Methods

### 4.1. Cell Culture, THC Exposure, and Sample Collection

Immortalized human bronchial epithelial cells (BEAS-2B) were obtained from the ATCC, Manassas, Virginia. The cells were grown in LHC-9 (1×) medium and maintained at 37 °C in a humidified incubator with 5% CO_2_ and 95% air in vented T-75 flasks (Corning, Durham, NC, USA). Delta-9-THC was purchased from Sigma Aldrich (T4764, St. Louis, MO, USA).

When cells were 95–100% confluent in the flask, 5000 cells were seeded in each well of 24-well plates and incubated until they became 80% confluent in each well. Next, we exposed the BEAS-2B cells to 800, 1000, 1200, or 1500 ng/mL of Δ-9-THC or a vehicle control (0 ng/mL) for 24 h. The cells were harvested after the 24 h exposure for endpoint measurement and downstream mechanistic analysis. The concentrations of Δ-9-THC (800, 1000, 1200, and 1500 ng/mL) were selected to encompass a range from sub-cytotoxic to cytotoxic levels in the BEAS-2B cell line, enabling the evaluation of dose-dependent cellular responses and the identification of threshold concentrations that trigger stress and cell death pathways. To investigate the effect of THC on cell growth and changes in global transcriptomics, 75% confluent cells were inoculated in fresh culture medium containing Δ-9-THC with three technical replicates.

### 4.2. Cell Viability Analysis

Cells were seeded into 96-well plates with 5000 cells per well. To determine the cell viability, BEAS-2B cells were exposed to a range of concentrations of Δ-9-THC (0.06–2400 ng/mL). The cell viability was determined using a 3-(4,5-dimethylthiazol-2-Yl)-2,5-diphenyltetrazolium bromide (MTT) cell proliferation assay (Thermo Fisher, Waltham, USA). Briefly, the MTT assay measures the conversion of MTT (3-(4,5-dimethylthiazol-2-Yl)-2,5-10 diphenyltetrazolium bromide) to an insoluble formazan. The MTT stock solution (2 mg/mL of PBS) was prepared and diluted to a 1:9 ratio with medium, and cells were incubated in it for two hours. After incubation, the MTT solution was decanted, and cells were rinsed with 1× PBS. Then, DMSO was added, and cells were scanned using a Biotek microplate reader at an absorbance of 540 nm.

### 4.3. RNA/DNA Extraction and cDNA Synthesis

According to the user’s guide, total RNA was extracted from the cells using a ZR Duet kit (Zymo Research, Irvine, CA, USA). Following extraction, cDNA was synthesized from 2 μg of total RNA per sample using a High-Capacity cDNA Reverse Transcription Kit (Applied Biosystems, Foster City, CA, USA). Oligo (dT) and random primer mixes were used following the guidelines provided by the user’s guide.

### 4.4. Library Preparation, RNA Sequencing, and Bioinformatics

The RNA sequencing library was prepared using the NEBNext^®^ Ultra II RNA Library Prep Kit for Illumina (E7775, NEB, Ipswich, MA, USA) according to the manufacturer’s instructions. RNA was then purified, fragmented, isolated, and primed for double-stranded cDNA synthesis. Sequence adaptors and indexes were then ligated to the end of the dscDNA sample. Polymerase chain reaction was then used to amplify the sample through 13 cycles. Quality control was then completed by cleaning up, assessing, and quantifying the amplified cDNA on a bioanalyzer. RNA sequencing was performed on the HiSeq X ten (Illumina) platform (150bpxPE) at Novogene Corporation (Davis, CA, USA) with a sequencing output of 40 million reads per sample. Bioinformatic analysis of the sequence data was performed using established pipelines. Cytoscape 3.10.3, Shiny GO 0.82, and STRING database 12.0; enrichment analyses using GO terms (http://www.geneontology.org/, accessed on 12 May 2024); and KEGG pathways (http://www.genome.jp/kegg/, accessed on 24 May 2024) were used for downstream transcriptome analysis and data visualization. KEGG pathways and GO keywords with *p*-values less than 0.05 were considered significantly enriched. For GO term analysis, a false discovery rate (FDR) cutoff 0.05 and recommended default background were used in Shiny GO. The DEGs enriched in the treated groups were obtained by comparison with the genes in the vehicle control group.

### 4.5. Pathway Analysis

Enrichment analysis of significant genes was performed using Gene Ontology, Ingenuity Pathway Analysis (IPA), and KEGG pathway analysis. IPA software 21.0 was also used to reveal the pathways that were most altered as a result of exposure to different concentrations. Overlapping DEGs between each treatment group were visualized by using Venn diagrams produced by Venny 2.1 (https://bioinfogp.cnb.csic.es/tools/venny/, accessed on 11 September 2024). Shiny GO [60] and Cytoscape [61] were used for identifying disease-specific pathways. The TLSEA was used for lncRNA set enrichment [62].

### 4.6. Gene Validation by Real-Time Quantitative PCR (qPCR)

RNA was isolated from cells and reverse transcribed to cDNA, as described above. Real-time qPCR was conducted using PowerUp SYBR Green PCR Master Mix (Applied Biosystems, Foster City, CA, USA) with a QuantStudio 3 (Applied Biosystems, Foster City, CA, USA). Real-time qPCR was performed using PowerUp SYBR TM Green PCR Master Mix (Applied Biosystems, Foster City, CA, USA) with QuantStudio 3 (Applied Biosystems, Foster City, CA, USA). Primers specific to ferroptosis (*HO1*, *FTH*, *FTL*, *CHAC1*, *GCLC* and *GSH*), unfolded protein response (*BIP*, *PERK*, *ELF2A*, and *SREPB*), the NRF2 pathway (*NRF2*, *SOD*, *CAT*, *GST*, and *SQSTM1*), oxidative stress (*SENS2*, *IL8*), DNA methylation (*DNMT* and *DNMT3A*), and a cannabinoid receptor (*CB1*) were examined.
HO1Forward: 5′ CTC TGA AGT TTA GGC CAT TG 3′ Reverse: 5′ AGT TGC TGT AGG GCT TTA TG 3′FTHForward: 5′ GCA CGA GCA AGT CAA GAC CAT 3′ Reverse: 5′ CTT GTC GAA CAG GTA CTC AGC 3′FTLForward: 5′ CAG CCT GGT CAA TTT GTA CCT 3′ Reverse: 5′ GCC AAT TCG CGG AAG AAG TG 3SQSTM1Forward: 5′ GCC ATA CCC TCT TCG ACT ACG 3′ Reverse: 5′ GAT TCT GGC ATC TGT AGG 3′PERKForward: 5′ CTT ATG CCA GAC ACA CAG AA 3′ Reverse: 5′ TCC ATC GTG CTG AAT GGA ATA C 3′

### 4.7. Testing for the Role of Ferroptosis in Cell Viability

To characterize the role of ferroptosis in Δ-9-THC treated cells, we examined the effects of an agonist and antagonist of ferroptosis on BEAS-2B cells exposed to 1200 ng/mL Δ-9-THC, which resulted in a significant reduction in cell viability. Cells were cultured as described above. When cells were 95–100% confluent, they were seeded in 24-well plates and exposed to Δ-9-THC, vehicle, ferrostatin-1 (an agonist, Fer-1, 5 µM), erastin (an antagonist, ERA, 1 µM), or ERA + Fer-1 for 24 h. The cells were harvested after the 24 h exposure. Delta-9-THC (T4764), ferrostatin-1 (SML0583), and erastin (E7781-1 mg) were purchased from Sigma Aldrich (St. Louis, MO, USA). Fer-1 and ERA were both dissolved in dimethyl sulfoxide (DMSO), and Fer-1 was applied 1 h before delta-9-THC exposure. A 3-(4,5-dimethylthiazol-2-Yl)-2,5-diphenyltetrazolium bromide (MTT) cell proliferation assay (Thermofisher, Waltham, MA, USA) was used to evaluate cell viability [63].

### 4.8. GSH Measurement

Cells were collected and re-suspended in an ice-cold 50 mm potassium phosphate buffer. The cell suspension was sonicated (provide parameters and time), followed by centrifugation at 13,000× *g* for 10 min at 4 °C to remove cell debris. The supernatant was then collected, and protein concentrations were quantified using a Bio-Rad protein assay (Bradford, Hercules, CA), using bovine serum albumin (BSA) as the standard. Protein lysate from treated cells was used to measure total cellular glutathione (GSH) according to established procedures [64]. Fluorescence intensity was measured at an excitation wavelength of 350 nm and an emission wavelength of 420 nm. The sample GSH content was calculated using a GSH (Sigma–Aldrich, St. Louis, MO, USA) standard curve and expressed as nanomoles of GSH per milligram of sample protein.

### 4.9. Intracellular ROS Measurement

Intracellular ROS was determined using an orange dye to quantify ROS. Cells were stained with the ROS orange working solution and incubated for 60 min at 37 °C. After incubation, cells were scanned in a Biotek microplate reader at an absorbance of 540/570 nm. Lipid peroxides were detected as malondialdehyde (MDA) reacted with thiobarbituric acid (TBA) to form a 1:2 adduct (color complex, TBARS) measurable by spectrofluorometric analysis at 530 nm. The concentrations of TBARS were calculated using MDA as a reference standard. The quantities of TBARS were expressed in terms of amount (pmol) per mg protein.

### 4.10. Statistical Analysis

One-way ANOVA was used to determine statistically significant differences in means among three or more groups. Following ANOVA, Dunnett’s post hoc test was applied when comparisons were made exclusively against a control group. If all group comparisons were of interest, Tukey’s HSD test was used instead. Statistical significance was defined as *p* < 0.05 unless otherwise stated. The cell viability, gene expression, and agonist/antagonist data were analyzed by one-way ANOVA. A *t*-test was used to compare two means.

## 5. Conclusions

In summary, the transcriptome analysis of bronchial epithelial cells exposed to Δ-9-THC revealed genes with distinctly altered patterns of expression, particularly those specific to ferroptosis, unfolded protein response, and tumor microenvironment pathways. The extent of gene deregulation was dependent on the concentration of Δ-9-THC. To our knowledge and based on the literature, the ferroptosis pathway is likely a novel pathway associated with Δ-9-THC-induced cytotoxicity in bronchial epithelial cells. The present findings also show that Fer-1, an inhibitor of ferroptosis, ameliorates Δ-9-THC-induced cell death in airway cells, suggesting that ferroptosis is one of the mechanisms behind cell death. While the HIF-1 pathway could also be of interest, especially given its relevance to hypoxic responses and inflammation, we prioritized ferroptosis due to its direct relevance to the viability outcomes central to our study. Future studies will address Δ-9-THC-induced epigenetic dysregulation and its potential role in the activation of ferroptosis and HIF-1 signaling pathways. Nutritional interventions aimed at modulating epigenetic alterations associated with Δ-9-THC-induced ferroptosis pathway regulation in human bronchial epithelial cells may offer a promising strategy to mitigate the adverse effects of Δ-9-THC on human lungs.

## Data Availability

The transcriptome data are available from NCBI GEO as dataset #GSE159489. All other data are presented in the manuscript. The raw data can be made available by the corresponding author upon written request.

## Supplementary Materials

The following supporting information can be downloaded at: https://www.mdpi.com/article/10.3390/ijms26115212/s1.

## Abbreviations

The following abbreviations are used in this manuscript:

THC	Delta-9-tetrahydrocannabinol
RNAseq	RNA sequencing
qPCR	Quantitative teal-time polymerase chain reaction
BEAS2B	Epithelial cells isolated from normal human bronchial epithelium derived from autopsies of noncancerous individuals
