# Supplementary material for: Cellular and Transcriptional Responses of Human Bronchial Epithelial Cells to Delta-9-Tetrahydrocannabinol In Vitro
Source: Int J Mol Sci. 2025 May 29;26(11):5212. doi: 10.3390/ijms26115212 (PMC12155351; doi:10.3390/ijms26115212)
Supplement: Supplementary file 1 [file ijms-26-05212-s001.zip › ijms-3579034-supplementary.pdf]

Supplementary Table S1

A list of top 10 dysregulated mRNAs by 24-hour Δ-9-THC exposure. Those indicating inf in the Log2 Fold Change column had zero value in control, suggesting that they were activated by THC exposure and may serve as biomarkers of THC exposure in bronchial cells.

		Downregulated					Upregulated		
Concentration		Gene symbol	Log2 Fold change	Q Value	Concentration		Gene symbol	Log2 Fold change	Q Value
800	1	MCRIP1	-4.58684	0.001878	800	1	ABR	4.92421	0.004818
	2	SSBP1	-4.53692	0.001878		2	FAM20C	4.37887	0.001878
	3	GFUS	-3.81228	0.001878		3	SLCO5A1	4.13916	0.001878
	4	MED18	-3.44421	0.030653		4	MMP1	3.81462	0.001878
	5	GBA	-3.39334	0.001878		5	ATF6B	3.5628	0.001878
	6	TADA2A	-3.37367	0.004818		6	SLC25A6	3.50842	0.001878
	7	TAF9	-3.36146	0.017186		7	NEDD8	3.39449	0.001878
	8	SLC16A1	-3.06372	0.001878		8	IL1RL1	3.18243	0.001878
	9	MRPS12	-3.06178	0.001878		9	C1RL	3.07876	0.014481
	10	SGPP1	-3.02411	0.008371		10	BRD2	3.0684	0.001878
1000	1	SLC39A4	-4.7043	0.006095	1000	1	MT1G	inf	0.001878
	2	GBA	-4.31396	0.001878		2	MMP1	6.28529	0.001878
	3	MCRIP1	-4.02778	0.001878		3	IL1RL1	6.12073	0.001878
	4	RPS25	-3.86789	0.001878		4	KLHDC7B	4.59062	0.008371
	5	FAN1	-3.76331	0.017186		5	LCN2	4.29486	0.001878
	6	PCK2	-3.67426	0.003423		6	RAD17	4.2562	0.011499
	7	GFUS	-3.54866	0.001878		7	HSPA6	3.95743	0.001878
	8	SLC25A24	-3.39671	0.006095		8	NEDD8	3.85935	0.001878
	9	EIF3K	-3.00309	0.001878		9	HMOX1	3.54616	0.001878
	10	DDT	-2.98129	0.001878		10	ZNF469	3.49049	0.001878
1200	1	SLC39A4	-5.29575	0.012503	1200	1	CDC42EP5	inf	0.001878
	2	ATOH8	-4.461	0.001878		2	MT1G	inf	0.001878
	3	NDUFS3	-3.81763	0.001878		3	RN7SKP11	inf	0.001878
	4	TNNI2	-3.42941	0.009452		4	MMP1	7.01104	0.001878
	5	ECH1	-3.37068	0.001878		5	IL1RL1	6.57479	0.001878
	6	DHRS4	-3.36445	0.004818		6	LCN2	4.88431	0.001878
	7	PEG10	-3.11481	0.001878		7	HSPA6	4.42459	0.001878
	8	PCDH18	-3.04253	0.001878		8	KLHDC7B	4.29577	0.012503
	9	TAF9	-3.01899	0.022339		9	NCF2	4.12027	0.004818
	10	RPL7A	-3.00786	0.001878		10	HMOX1	4.07414	0.001878
1500	1	SLC39A4	-4.36553	0.001878	1500	1	LOC646938	inf	0.026199
	2	GFUS	-4.0102	0.001878		2	TNKS2-AS1	inf	0.018979
	3	MTCO2P12	-3.50199	0.02154		3	RPS27P9	inf	0.017186
	4	SYT8	-3.24789	0.001878		4	MT1G	inf	0.001878
	5	SURF2	-3.19974	0.009452		5	SPRR1B	inf	0.001878
	6	KIFC1	-3.17516	0.001878		6	RPS18	8.50609	0.022339
	7	LY6E	-2.93844	0.001878		7	HSPA6	7.77894	0.001878
	8	ANKRD2	-2.81355	0.011499		8	MMP1	5.43214	0.001878
	9	SLC25A24	-2.74909	0.001878		9	HSPA1B	5.16909	0.001878
	10	TNNI2	-2.68063	0.001878		10	HMOX1	4.82331	0.001878

Supplementary Figure S1

A

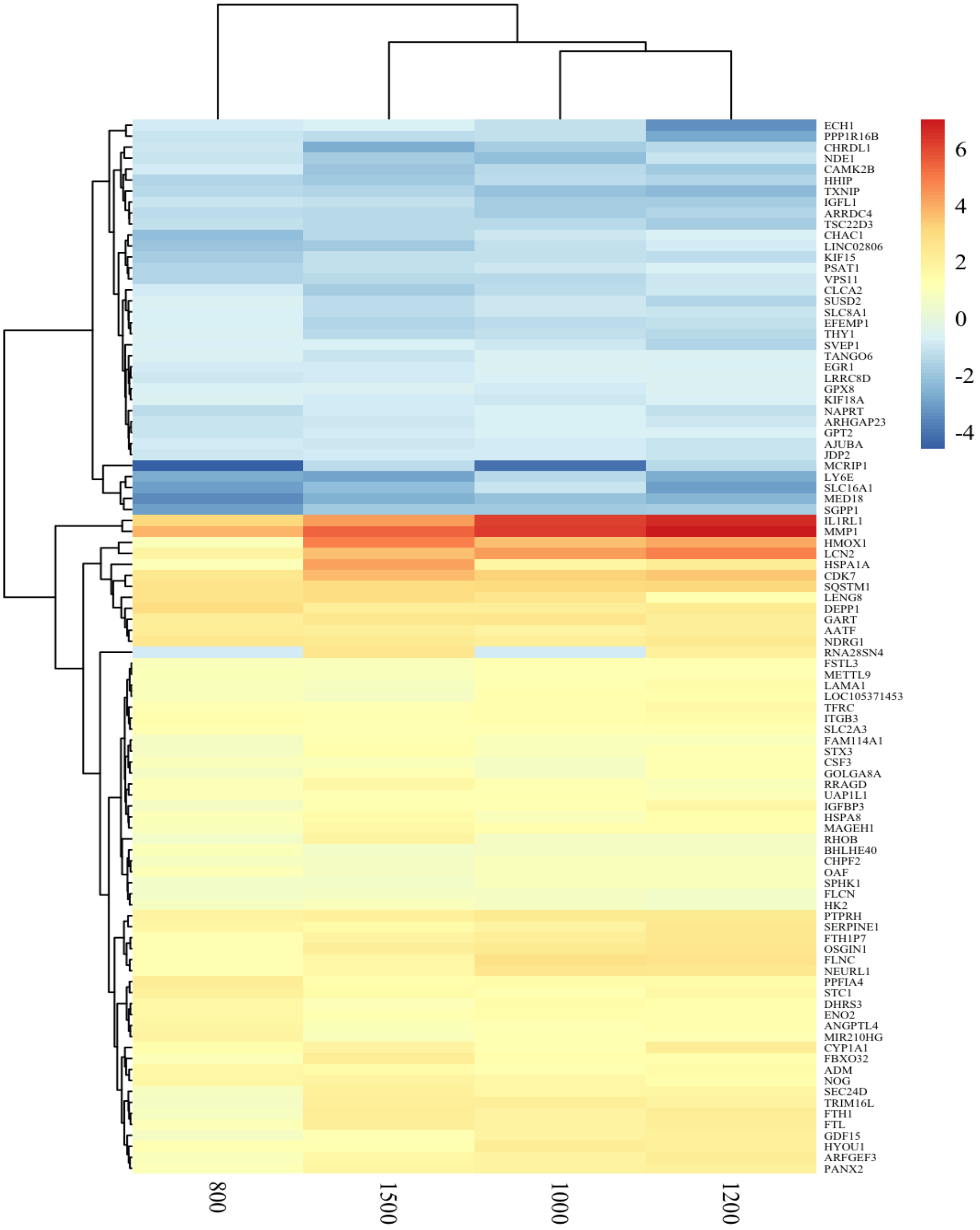

Figure S1. (A) Global alteration of gene expression profiles in BEAS-2B cells associated with all treatment groups of Δ-9-THC compared to the vehicle

B. Genes in various pathways

Ferroptosis-related genes

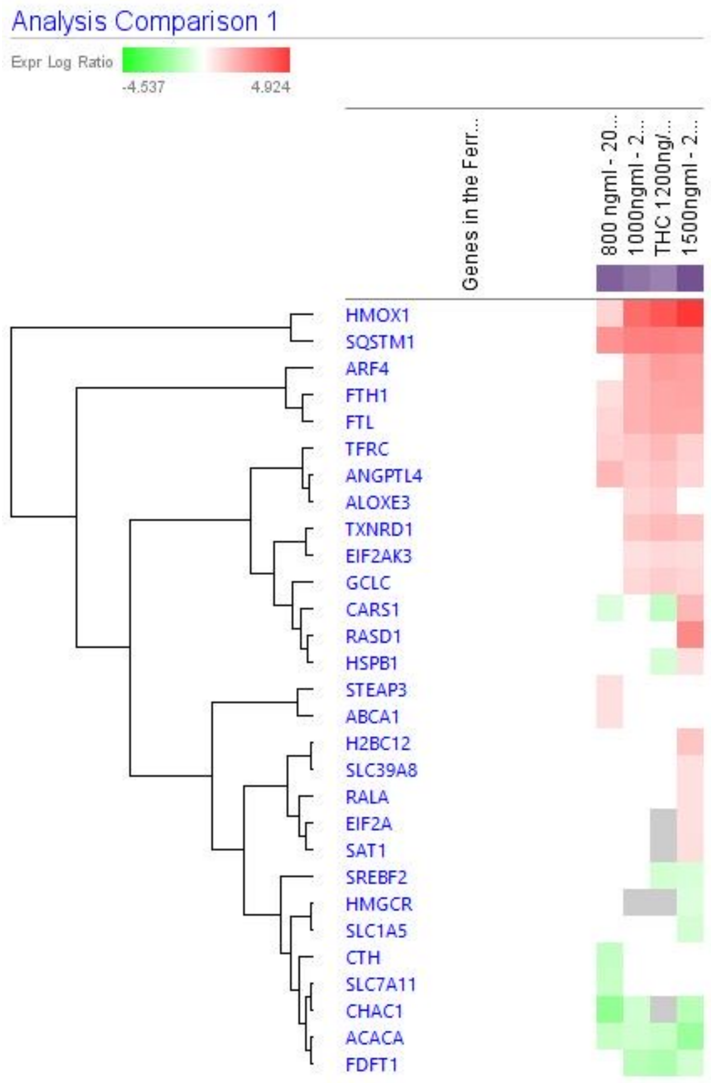

NRF2-related genes

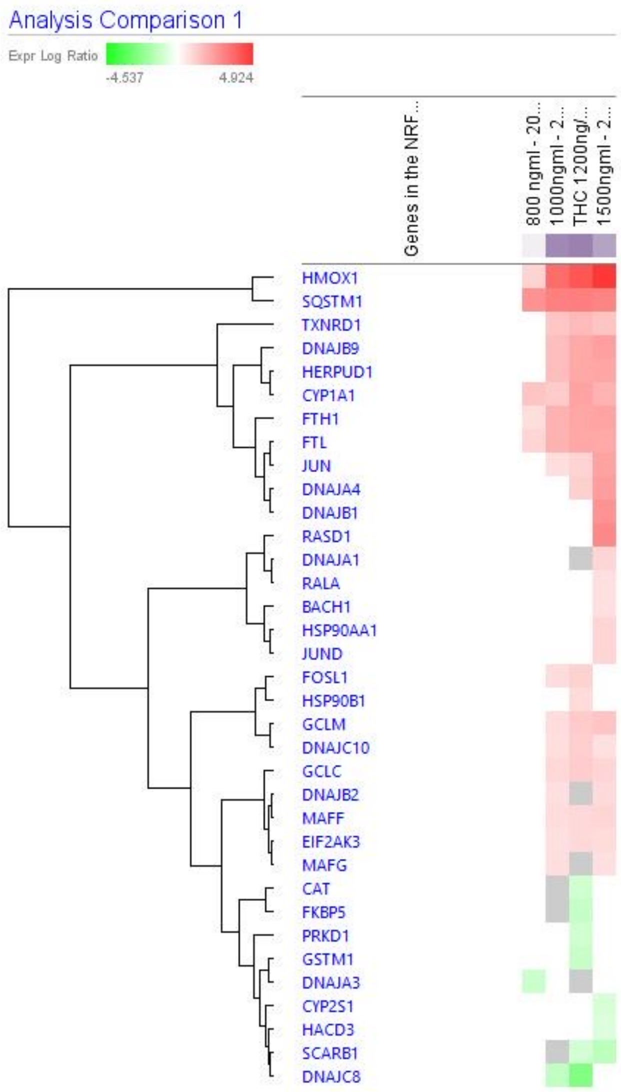

Figure S1. (B) Expression pattern of genes in the RNAseq database of THC-exposed BEAS-2B cells. A. A heatmap showing differentially regulated genes (upregulated and downregulated) in all treatment groups (FDR <0.1). B. Ferroptosis and NRF-related genes differentially expressed in THC-exposed cells. Data suggest that THC can causes alterations in the expression of genes involved in ferroptosis and NRF-2 pathway.

Supplementary Figure S2A

Enrichment FDR	Pathway Genes	Fold Enrichment	Pathway
0.002451271	33	138.1575758	Fructose and mannose metabolism
0.013422149	109	41.82752294	HIF-1 signaling pathway
0.019724723	5	455.92	Neomycin, kanamycin and gentamicin biosynthesis
0.033274529	246	18.53333333	Shigellosis
0.047199405	20	113.98	Glycosaminoglycan biosynthesis
0.049466222	67	34.0238806	Glycolysis / Gluconeogenesis
0.049466222	31	73.53548387	Galactose metabolism
0.049466222	35	65.13142857	Starch and sucrose metabolism
0.049466222	49	46.52244898	Amino sugar and nucleotide sugar metabolism
0.049466222	1527	4.478585462	Metabolic pathways
0.049466222	37	61.61081081	Biosynthesis of nucleotide sugars
0.049466222	72	31.66111111	Mitophagy
0.049466222	46	49.55652174	Type II diabetes mellitus
0.049466222	47	48.50212766	Carbohydrate digestion and absorption
0.049466222	57	39.99298246	Legionellosis
0.049466222	67	34.0238806	Acute myeloid leukemia
0.049466222	70	32.56571429	Central carbon metabolism in cancer
0.064529653	100	22.796	AGE-RAGE signaling pathway in diabetic complications
0.069418479	115	19.8226087	Carbon metabolism
0.069418479	120	18.99666667	AMPK signaling pathway
0.070635514	131	17.40152672	FoxO signaling pathway
0.070635514	141	16.16737589	Autophagy
0.070635514	137	16.63941606	Insulin signaling pathway
0.074356038	162	14.07160494	JAK-STAT signaling pathway
0.074356038	161	14.15900621	MicroRNAs in cancer
0.100994457	232	9.825862069	Thermogenesis

Figure S2. (A) Upregulated gene associated Pathways enrichment in BEAS-2B cells exposed to delta-9 THC *in vitro.* Metabolic pathways were the most dysregulated pathways followed by immune and one carbon metabolism pathways.

Supplementary Figure S2B

Enrichment FDR	Pathway Genes	Fold Enrichment	Pathway
1.28E-06	75	93.5220513	Biosynthesis of amino acids
0.000293	115	45.7444816	Carbon metabolism
0.0009831	37	94.7858628	Alanine, aspartate and glutamate metabolism
0.0009831	40	87.6769231	Glycine, serine and threonine metabolism
0.0037203	1527	5.74177623	Metabolic pathways
0.0303011	22	79.7062937	Arginine biosynthesis
0.0303011	19	92.291498	2-Oxocarboxylic acid metabolism
0.0408464	34	51.5746606	RNA polymerase
0.0500746	50	35.0707692	Cysteine and methionine metabolism
0.0500746	57	30.7638327	Glutathione metabolism
0.0500746	61	28.7465322	Arachidonic acid metabolism
0.0500746	63	27.8339438	Cytosolic DNA-sensing pathway
0.0548541	75	23.3805128	Thyroid hormone synthesis
0.1030885	155	11.3131514	Biosynthesis of cofactors
0.1826455	306	5.73051785	Huntington disease

Figure S2. (B) Downregulated gene associated Pathways enrichment in BEAS-2B cells exposed to delta-9 THC *in vitro.* Metabolic pathways were the most dysregulated pathways followed by immune and one carbon metabolism pathways.

Supplementary Figure S3

A

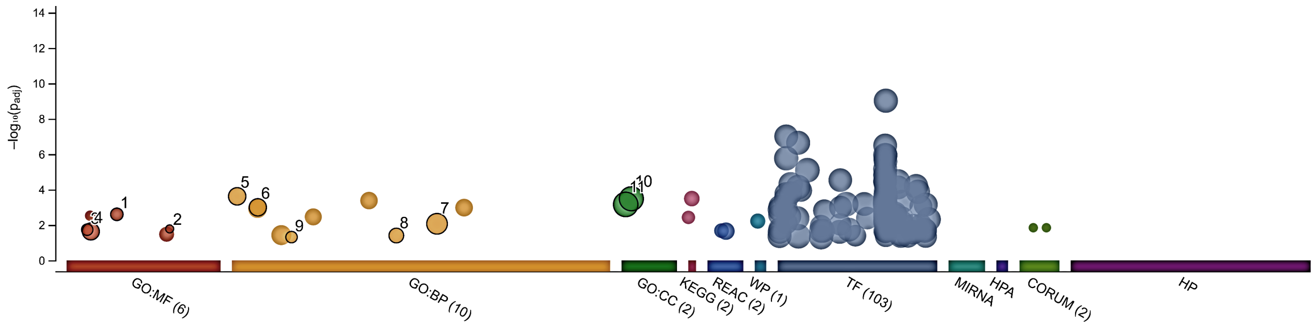

ID	Source	Term ID	Term Name	p_{adj} (query_1)
1	GO:MF	GO:0019200	carbohydrate kinase activity	2.503×10^{-3}
2	GO:MF	GO:0050196	[phosphorylase] phosphatase activity	1.665×10^{-2}
3	GO:MF	GO:0005536	glucose binding	1.844×10^{-2}
4	GO:MF	GO:0008514	organic anion transmembrane transporter activity	2.330×10^{-2}
5	GO:BP	GO:0001666	response to hypoxia	2.408×10^{-4}
6	GO:BP	GO:0005996	monosaccharide metabolic process	1.004×10^{-3}
7	GO:BP	GO:0055085	transmembrane transport	8.583×10^{-3}
8	GO:BP	GO:0045453	bone resorption	3.946×10^{-2}
9	GO:BP	GO:0010635	regulation of mitochondrial fusion	4.850×10^{-2}
10	GO:CC	GO:0016020	membrane	3.293×10^{-4}
11	GO:CC	GO:0005737	cytoplasm	6.918×10^{-4}

B

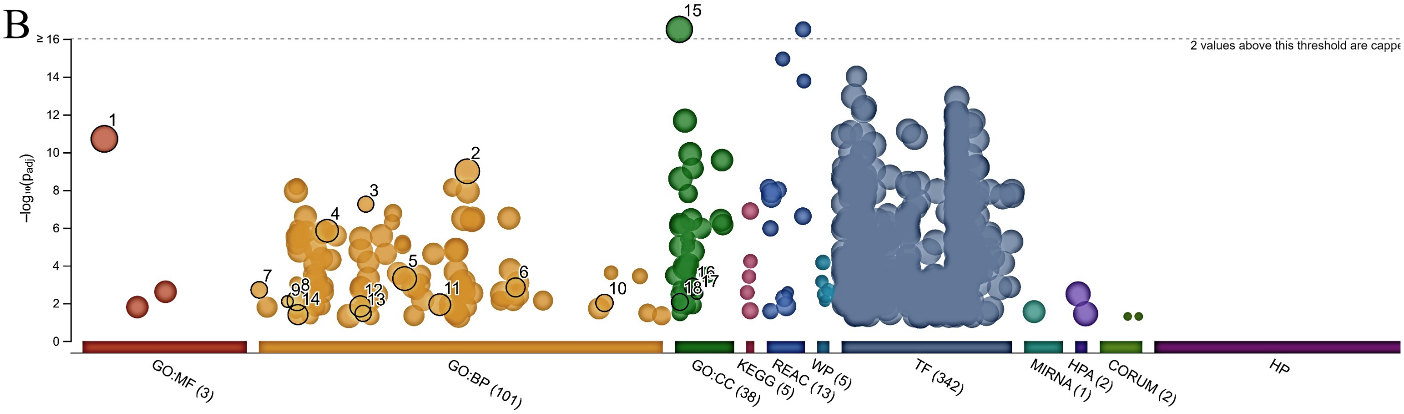

ID	Source	Term ID	Term Name	p_{adj} (query_1)
1	GO:MF	GO:0005515	protein binding	1.984×10^{-11}
2	GO:BP	GO:0051179	localization	1.028×10^{-9}
3	GO:BP	GO:0030968	endoplasmic reticulum unfolded protein response	5.690×10^{-8}
4	GO:BP	GO:0012501	programmed cell death	1.450×10^{-6}
5	GO:BP	GO:0036211	protein modification process	4.969×10^{-4}
6	GO:BP	GO:0071496	cellular response to external stimulus	1.448×10^{-3}
7	GO:BP	GO:0000041	transition metal ion transport	1.957×10^{-3}
8	GO:BP	GO:0006914	autophagy	7.292×10^{-3}
9	GO:BP	GO:0006048	UDP-N-acetylglucosamine biosynthetic process	8.135×10^{-3}
10	GO:BP	GO:1903008	organelle disassembly	9.641×10^{-3}
11	GO:BP	GO:0045944	positive regulation of transcription by RNA polym...	1.186×10^{-2}
12	GO:BP	GO:0022603	regulation of anatomical structure morphogenesis	1.469×10^{-2}
13	GO:BP	GO:0030433	ubiquitin-dependent ERAD pathway	3.452×10^{-2}
14	GO:BP	GO:0007005	mitochondrion organization	4.016×10^{-2}
15	GO:CC	GO:0005737	cytoplasm	3.244×10^{-17}
16	GO:CC	GO:0031985	Golgi cisterna	1.325×10^{-3}
17	GO:CC	GO:0034663	endoplasmic reticulum chaperone complex	2.960×10^{-3}
18	GO:CC	GO:0005776	autophagosome	8.611×10^{-3}

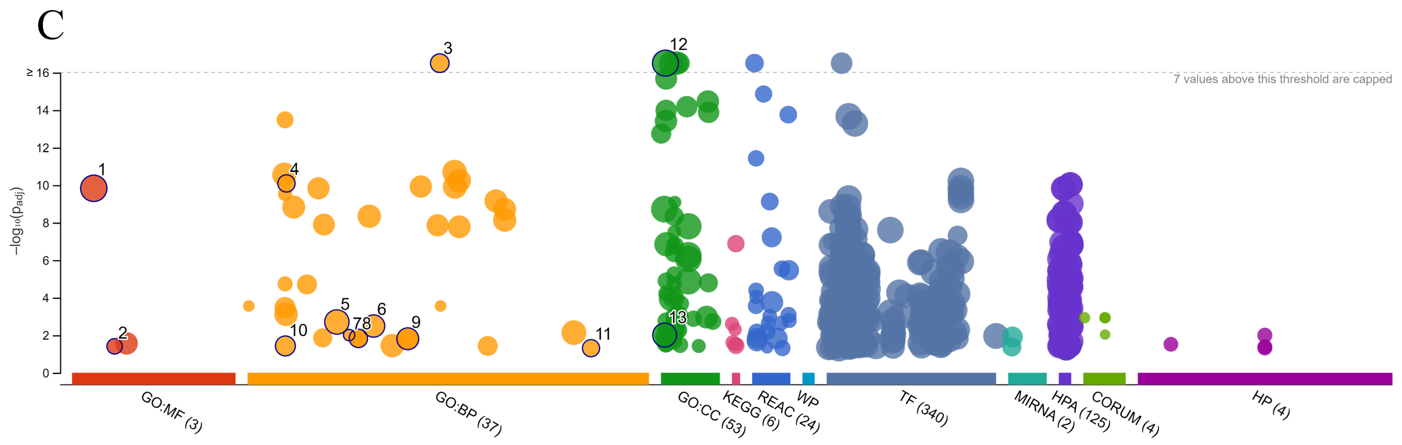

ID	Source	Term ID	Term Name	p_{adj} (query_1)
1	GO:MF	GO:0005515	protein binding	1.483×10^{-10}
2	GO:MF	GO:0016706	2-oxoglutarate-dependent dioxygenase activity	3.909×10^{-2}
3	GO:BP	GO:0048193	Golgi vesicle transport	6.508×10^{-17}
4	GO:BP	GO:0007030	Golgi organization	8.148×10^{-11}
5	GO:BP	GO:0019538	protein metabolic process	1.966×10^{-3}
6	GO:BP	GO:0033554	cellular response to stress	3.229×10^{-3}
7	GO:BP	GO:0030070	insulin processing	9.824×10^{-3}
8	GO:BP	GO:0031668	cellular response to extracellular stimulus	1.470×10^{-2}
9	GO:BP	GO:0043067	regulation of programmed cell death	1.542×10^{-2}
10	GO:BP	GO:0006914	autophagy	3.718×10^{-2}
11	GO:BP	GO:1903008	organelle disassembly	4.903×10^{-2}
12	GO:CC	GO:0005737	cytoplasm	1.507×10^{-27}
13	GO:CC	GO:0005654	nucleoplasm	9.822×10^{-3}

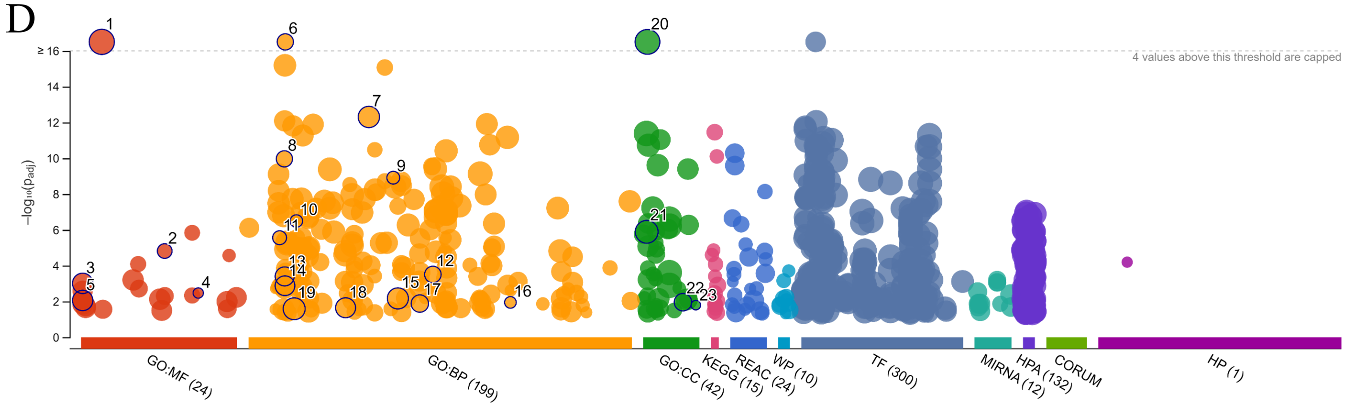

ID	Source	Term ID	Term Name	p_{adj} (query_1)
1	GO:MF	GO:0005515	protein binding	2.283×10^{-20}
2	GO:MF	GO:0044183	protein folding chaperone	1.533×10^{-5}
3	GO:MF	GO:0000987	cis-regulatory region sequence-specific DNA bindi...	9.792×10^{-4}
4	GO:MF	GO:0055131	C3HC4-type RING finger domain binding	3.392×10^{-3}
5	GO:MF	GO:0000981	DNA-binding transcription factor activity, RNA pol...	8.847×10^{-3}
6	GO:BP	GO:0006986	response to unfolded protein	9.056×10^{-18}
7	GO:BP	GO:0033554	cellular response to stress	4.942×10^{-13}
8	GO:BP	GO:0006888	endoplasmic reticulum to Golgi vesicle-mediated t...	1.084×10^{-10}
9	GO:BP	GO:0042026	protein refolding	1.223×10^{-9}
10	GO:BP	GO:0009226	nucleotide-sugar biosynthetic process	3.241×10^{-7}
11	GO:BP	GO:0006458	'de novo' protein folding	2.760×10^{-6}
12	GO:BP	GO:0048284	organelle fusion	3.128×10^{-4}
13	GO:BP	GO:0006914	autophagy	4.012×10^{-4}
14	GO:BP	GO:0006954	inflammatory response	1.332×10^{-3}
15	GO:BP	GO:0042592	homeostatic process	6.797×10^{-3}
16	GO:BP	GO:0090084	negative regulation of inclusion body assembly	1.136×10^{-2}
17	GO:BP	GO:0045926	negative regulation of growth	1.318×10^{-2}
18	GO:BP	GO:0030097	hemopoiesis	2.260×10^{-2}
19	GO:BP	GO:0009056	catabolic process	2.573×10^{-2}
20	GO:CC	GO:0005737	cytoplasm	3.945×10^{-21}
21	GO:CC	GO:0005654	nucleoplasm	1.290×10^{-6}
22	GO:CC	GO:0090575	RNA polymerase II transcription regulator complex	1.106×10^{-2}
23	GO:CC	GO:1990131	Gtr1-Gtr2 GTPase complex	1.617×10^{-2}

Figure S3. The GO terms represented by the genes dysregulated by THC exposure *in vitro*.

Supplementary Figure S4

A

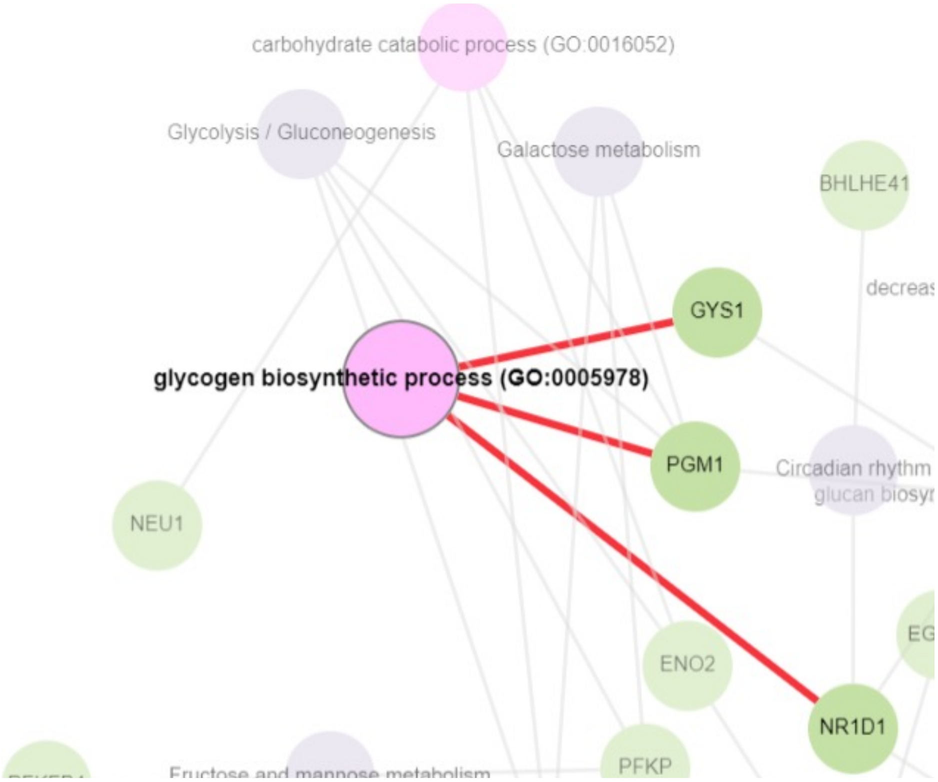

B

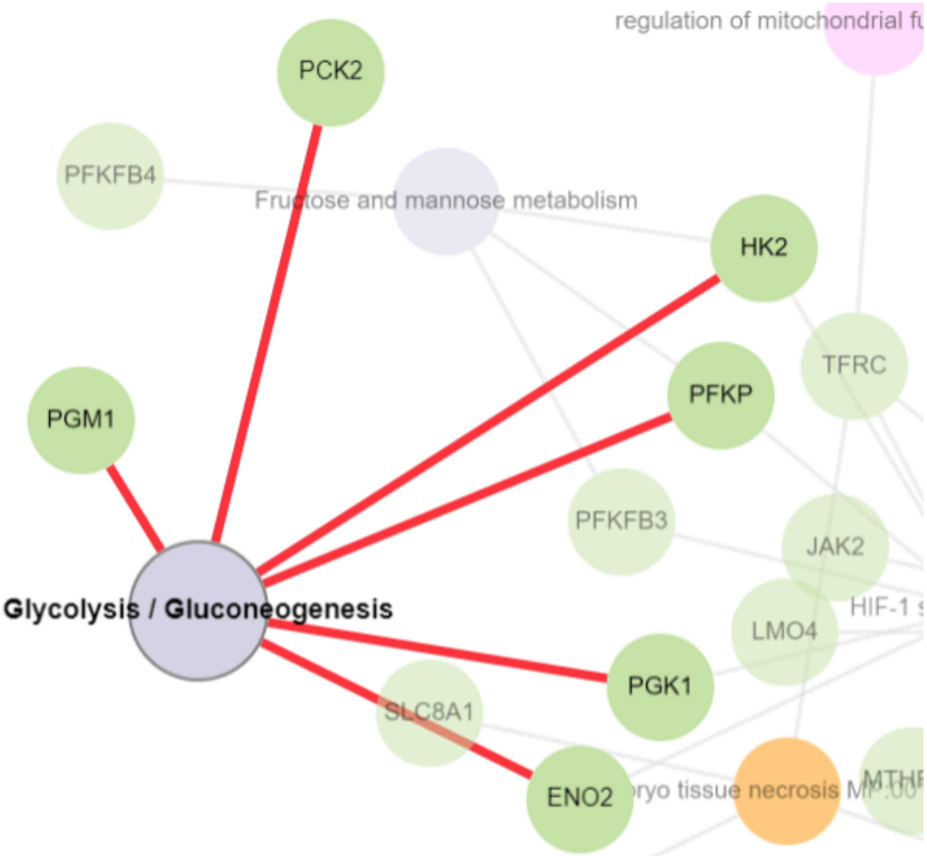

C

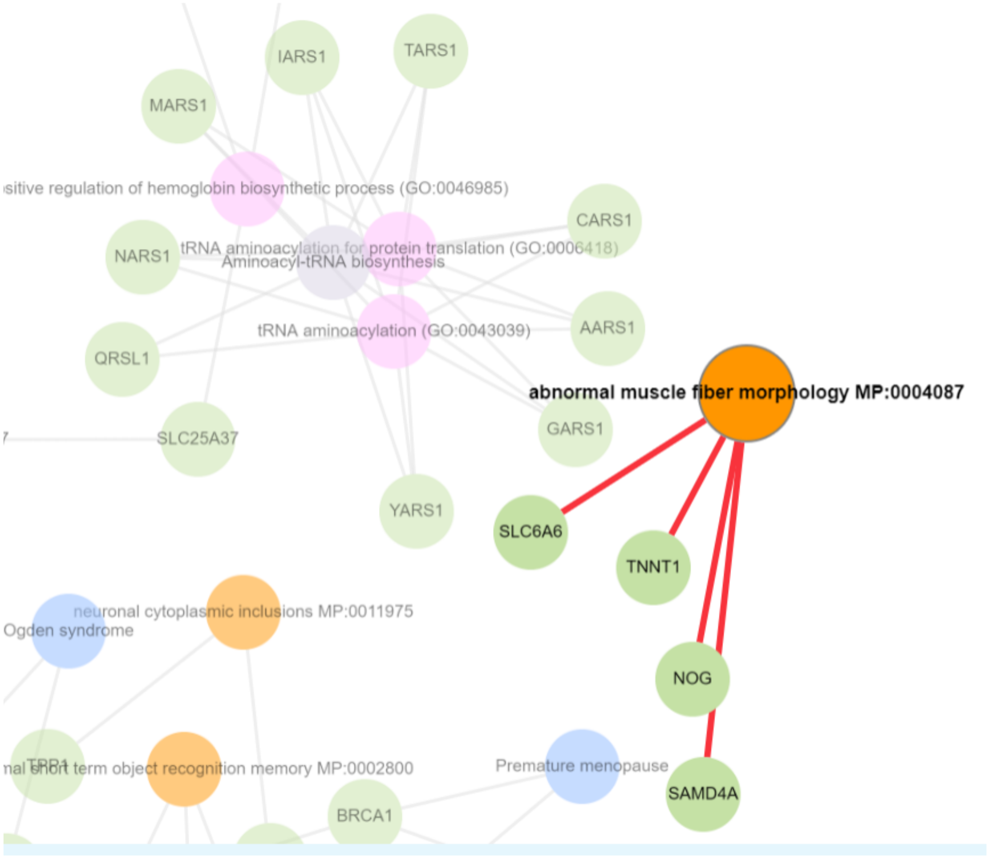

Figure S4. Major biological processes and their associated genes affected by THC exposure (800 ng/mL) *in vitro*.

Supplementary Figure S5

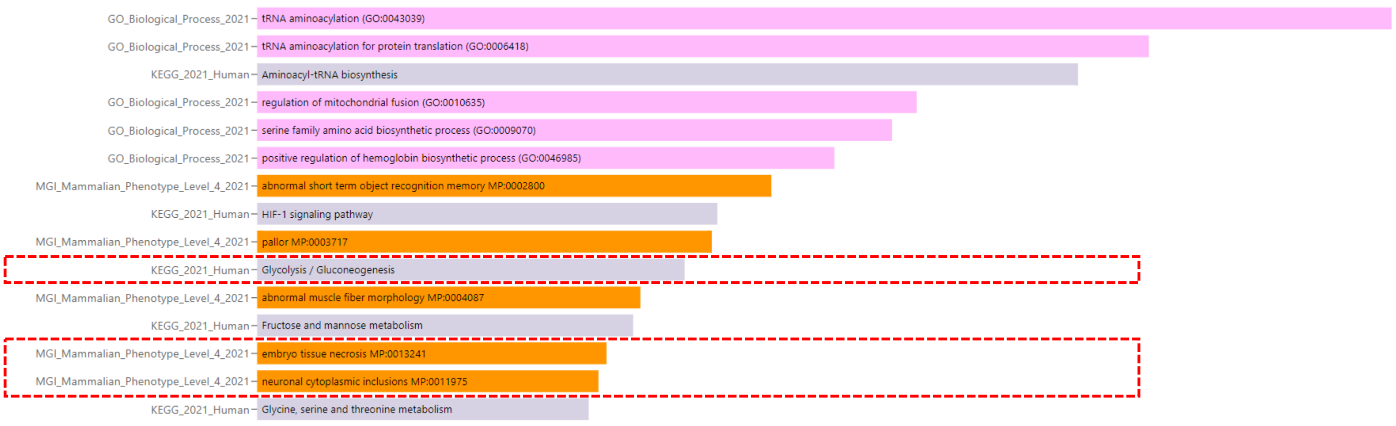

Figure S5. Pathways dysregulated by THC exposure (800 ng/mL) *in vitro.*

Supplementary Figure S6

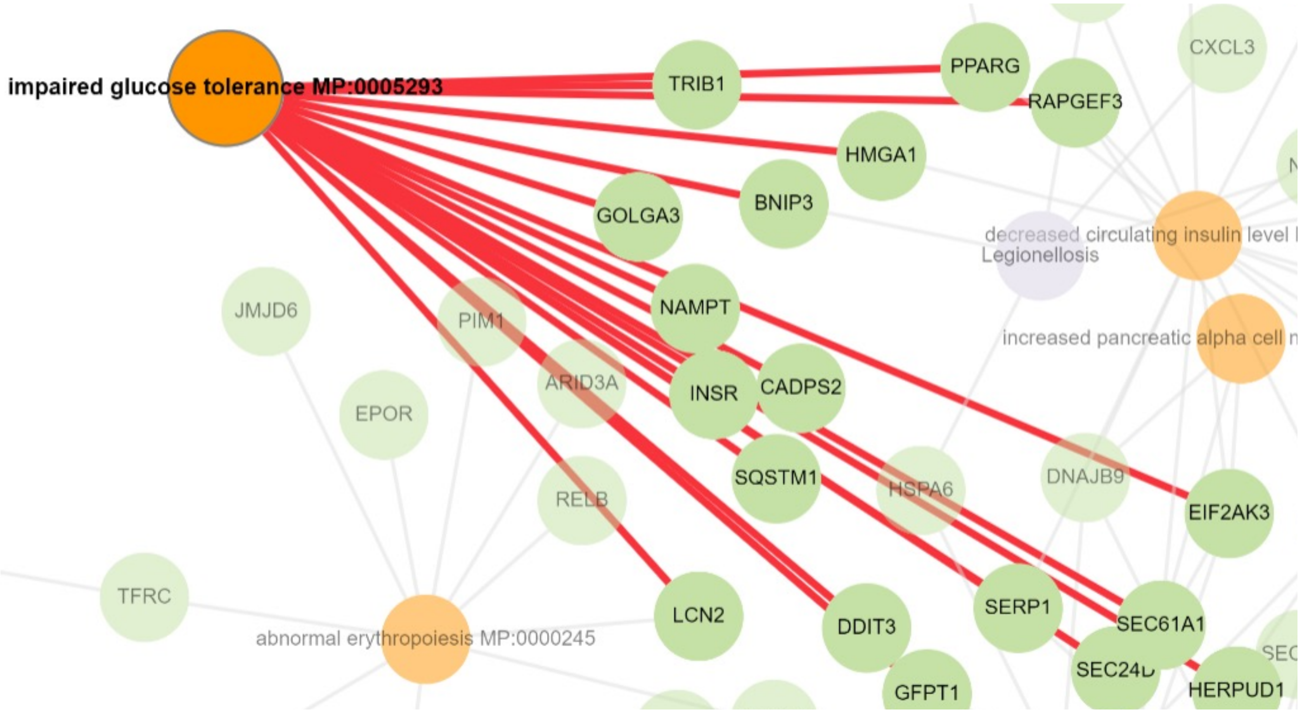

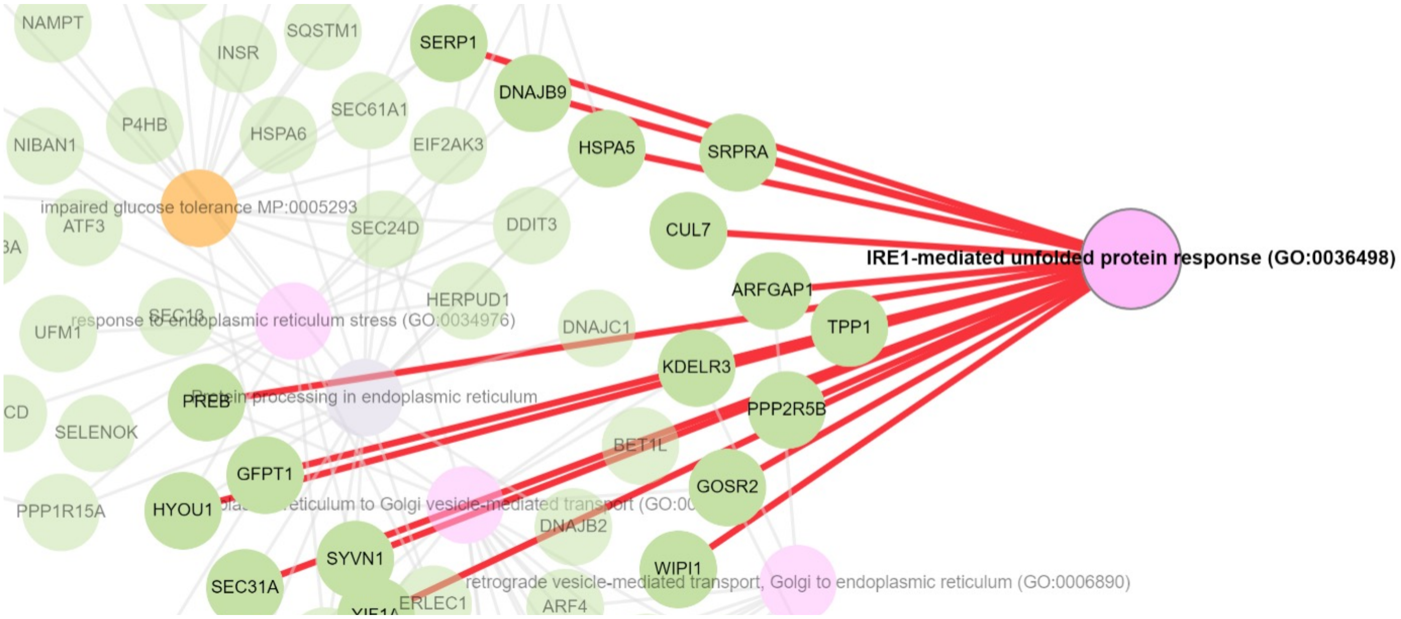

Figure S6. Impaired glucose response and IRE-1 mediated unfolded protein response dysregulated by THC exposure (1000 ng/mL) *in vitro*.

Supplementary Figure S7

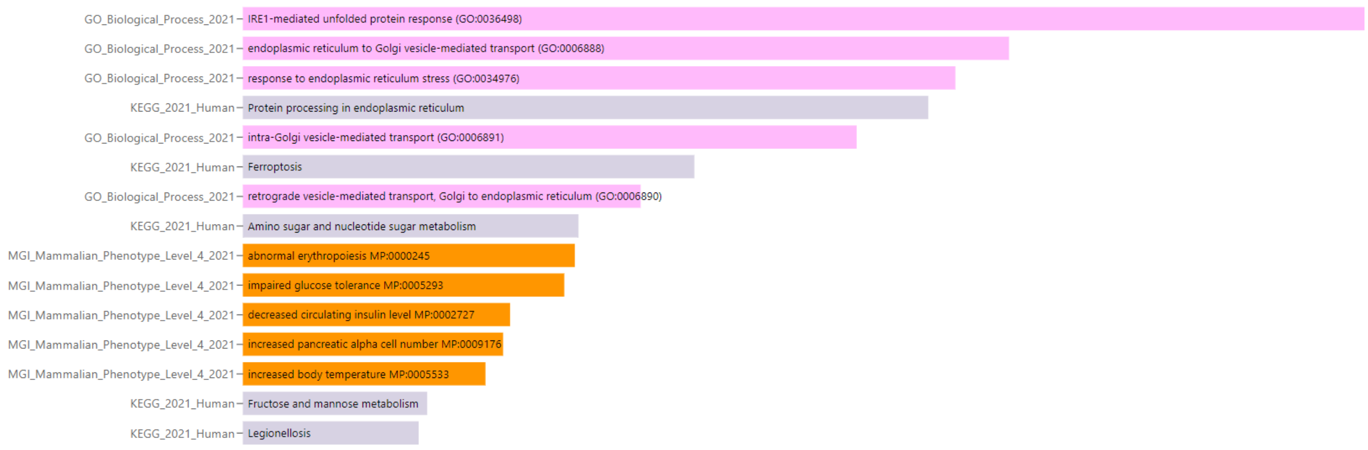

Figure S7. Pathways dysregulated by THC exposure (1000 ng/mL) *in vitro*.

Supplementary Figure S8

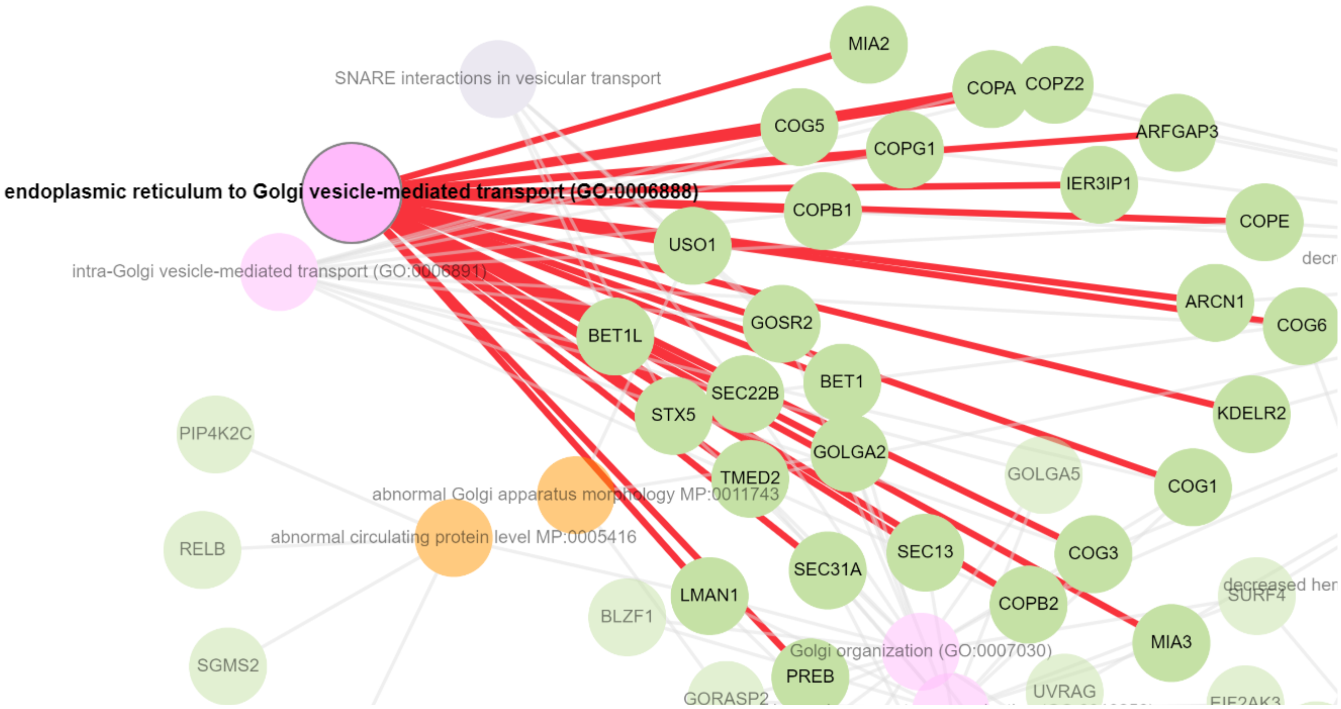

Figure S8. Genes most significantly dysregulated by THC exposure (1200 ng/mL) *in vitro*.

Supplementary Figure S9

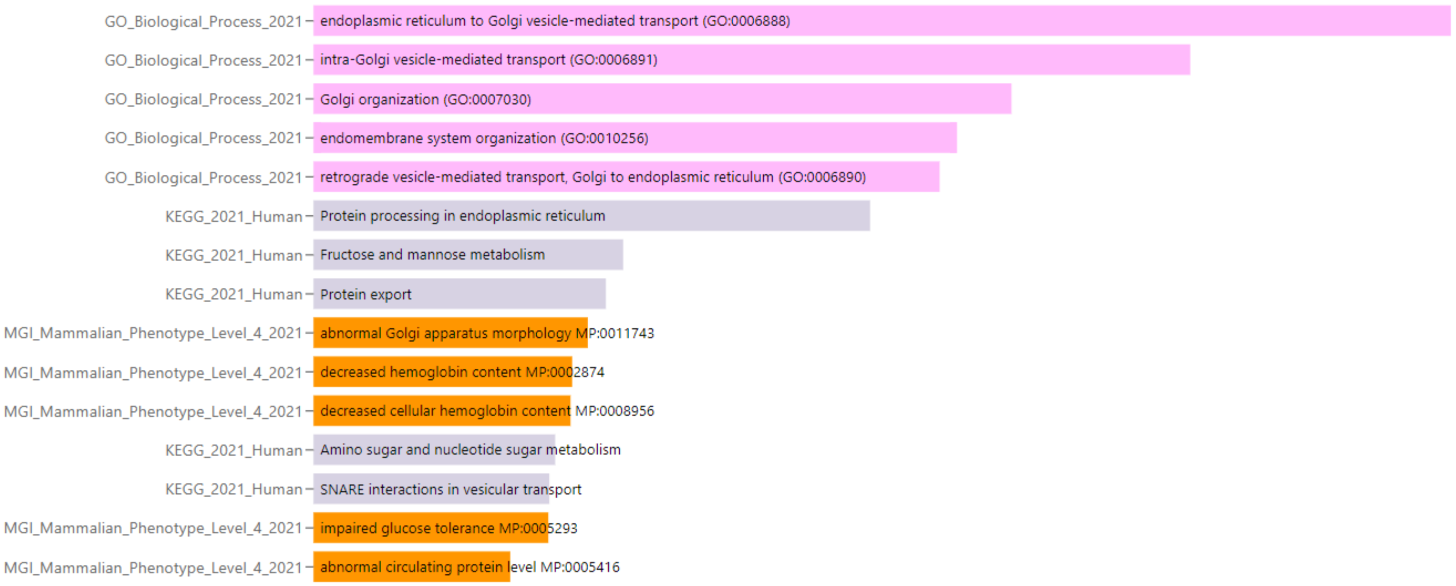

Figure S9. Pathways dysregulated by THC exposure (1200 ng/mL) *in vitro*.

Supplementary Figure S10

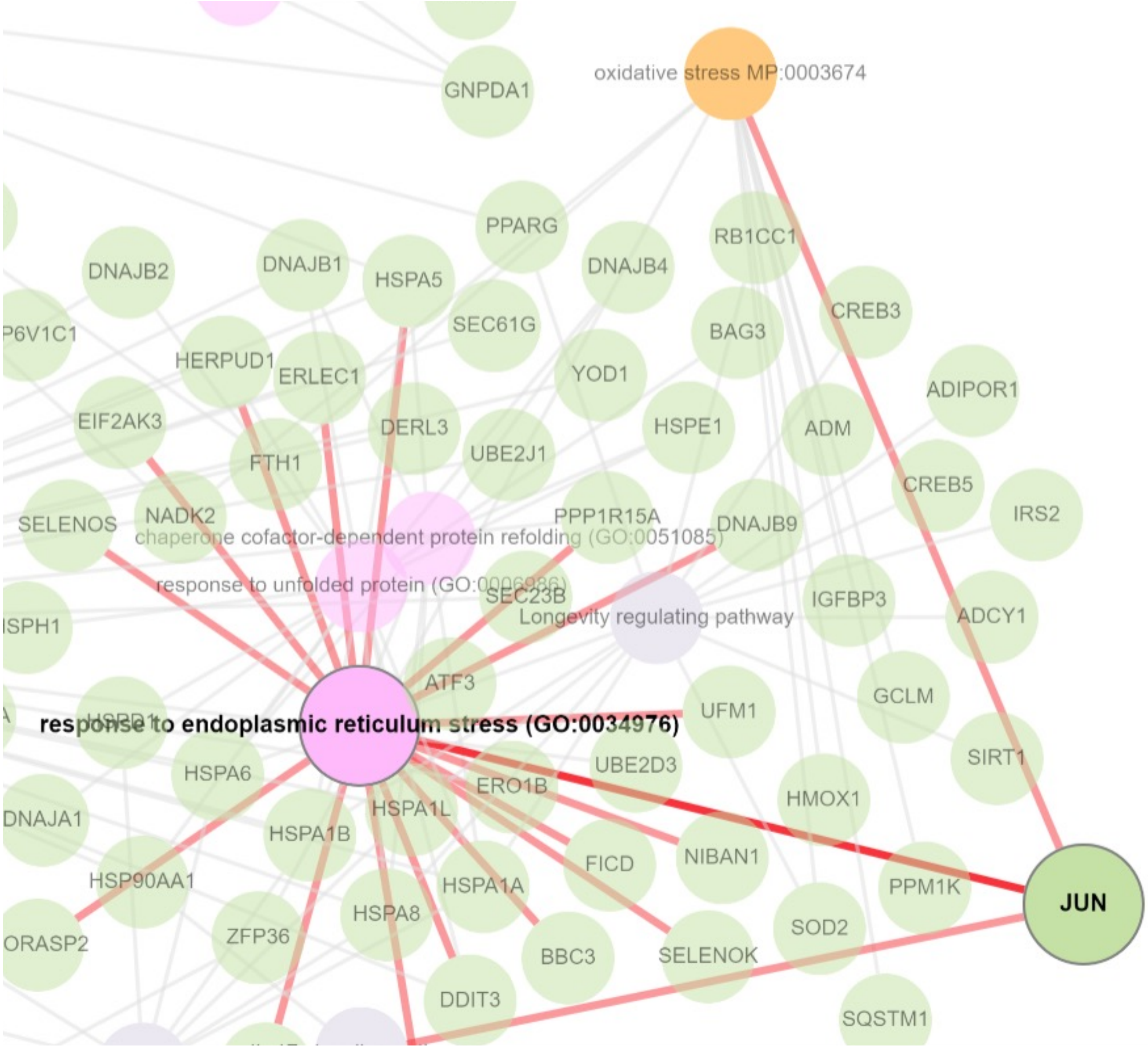

Figure S10. Pathways dysregulated by THC exposure (1500 ng/mL) *in vitro*.
